# Multifunctional GelMA platforms with nanomaterials for advanced tissue therapeutics

**DOI:** 10.1016/j.bioactmat.2021.06.027

**Published:** 2021-07-06

**Authors:** Amal George Kurian, Rajendra K. Singh, Kapil D. Patel, Jung-Hwan Lee, Hae-Won Kim

**Affiliations:** a Institute of Tissue Regeneration Engineering (ITREN), Dankook University, Cheonan, 31116, Republic of Korea; b Department of Nanobiomedical Science & BK21 NBM Global Research Center for Regenerative Medicine, Dankook University, Cheonan, 31116, Republic of Korea; c Biomaterials and Tissue Engineering, UCL Eastman Dental Institute, London, WC1X8LD, UK; d Department of Biomaterials Science, School of Dentistry, Dankook University, Cheonan, 31116, Republic of Korea; e UCL Eastman-Korea Dental Medicine Innovation Centre, Dankook University, Cheonan, 31116, Republic of Korea; f Cell & Matter Institute, Dankook University, Cheonan, 31116, Republic of Korea; g Department of Regenerative Dental Medicine, College of Dentistry, Dankook University, Cheonan, 31116, Republic of Korea; h Mechanobiology Dental Medicine Research Center, Dankook University, Cheonan, 31116, Republic of Korea

**Keywords:** GelMA hydrogel, Nanomaterials, Multifunctional, Therapeutics, Tissue repair

## Abstract

Polymeric hydrogels are fascinating platforms as 3D scaffolds for tissue repair and delivery systems of therapeutic molecules and cells. Among others, methacrylated gelatin (GelMA) has become a representative hydrogel formulation, finding various biomedical applications. Recent efforts on GelMA-based hydrogels have been devoted to combining them with bioactive and functional nanomaterials, aiming to provide enhanced physicochemical and biological properties to GelMA. The benefits of this approach are multiple: i) reinforcing mechanical properties, ii) modulating viscoelastic property to allow 3D printability of bio-inks, iii) rendering electrical/magnetic property to produce electro-/magneto-active hydrogels for the repair of specific tissues (e.g., muscle, nerve), iv) providing stimuli-responsiveness to actively deliver therapeutic molecules, and v) endowing therapeutic capacity in tissue repair process (e.g., antioxidant effects). The nanomaterial-combined GelMA systems have shown significantly enhanced and extraordinary behaviors in various tissues (bone, skin, cardiac, and nerve) that are rarely observable with GelMA. Here we systematically review these recent efforts in nanomaterials-combined GelMA hydrogels that are considered as next-generation multifunctional platforms for tissue therapeutics. The approaches used in GelMA can also apply to other existing polymeric hydrogel systems.

## Highlights

• Physicochemical properties of GelMA hydrogel.
• Role of nanomaterials for engineering bio-functional GelMA hydrogel.
• Diverse biomedical applications of nanostructured GelMA hydrogel.
• Promising directions for nano-inspired GelMA bioink formulation.
• Applications and challenges of advanced GelMA-nanomaterial platforms.

## Graphical abstract

**Figure:**
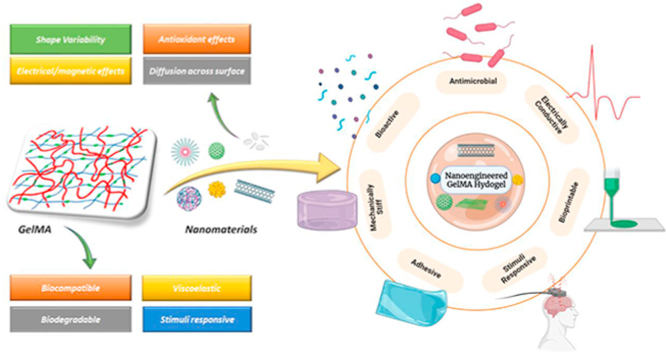

## 1 Introduction

Multifunctional hydrogels represent important components for engineering damaged tissues. Compared to other biomaterial platforms used for regenerative purposes, hydrogels have an increasing demand owing to their close resemblance to the cellular microenvironment [[1], [2], [3], [4], [5], [6], [7], [8], [9], [10]]. These materials have been actively examined so far to develop various biological substitutes and to rouse the predictable biological responses [6,[11], [12], [13], [14], [15], [16], [17], [18], [19]]. Regardless of these significant properties exhibited by the hydrogels, they still possess many limitations such as low mechanical stiffness, low thermal stability, fast degradation rates, etc. which restricts their efficient utilization for various applications [12,15,20,21]. Here comes the significance of nano-engineered hydrogel systems. These hydrated polymeric networks with either covalently or non-covalently ingrained nanomaterials can behave as multi-responsive platforms that mimic the native cellular matrix and possesses regenerative properties for engineering impaired tissues. These hybrid nano-biomaterials also exhibit better biocompatibility, good cellular viability, proliferation, and differentiation in response to cells [[22], [23], [24], [25], [26], [27], [28]].

Bulcke et al. synthesized one such representative hydrogel formulation in the year 2000 popularly known as Gelatin methacryloyl or GelMA [29]. GelMA owns common arginyl-glycyl-aspartic acid (RGD), a tripeptide that favors certain cellular activities such as attachment, spreading, and differentiation into various lineages, and matrix metalloproteinase (MMP) sequences belonging to endopeptidases which support enzymatic degradation and play key roles in dermal wound healing, morphogenesis, and tissue restoration [[30], [31], [32], [33], [34], [35], [36]]. Thus this photoreactive gelatin derivative has been extensively used for various biological applications from simple cell culture scaffolds to intricate tissue engineering platforms and advanced drug, gene, or growth factor delivery vehicles [31,37,38]. Current research interests utilize incorporating different nanomaterials into the GelMA network to obtain nano-structured hydrogels. The interaction of nanophase with the hydrogel structure results in the characteristic properties of the hybrid hydrogels which lack in individual constituents [22,27,39,40]. Nanomaterial incorporation could reinforce GelMA and offers responsiveness to external stimuli such as thermal, electromagnetic and, mechanical in particular. The nature of nanomaterials incorporated into the hydrogel also governs the kind of stimuli to which its hybrid form is receptive [[41], [42], [43], [44], [45], [46], [47], [48], [49], [50], [51]]. Apart from this, the introduction of nanomaterials into hydrogels also improves their injectability and shear thinning properties. The native cellular matrix is known to be viscoelastic and is responsive to various mechanical stimuli. A good performance injectable hydrogel is expected to have suitable rheological features, dimensional integrity, and mechanical properties. In most of the nanoengineered injectable hydrogels, the nanomaterial present modulates rheological response by unique interactions and instills better viscoelastic characteristics (increased shear moduli and relative elasticity) to the overall system which results from the porous microarchitecture of hydrogels and various interactions that arise between hydrophobic polymer chains and nanomaterials [52]. The permeability of hydrogels is also affected by such factors [53]. The concept of hydrogel permeability possesses clinical significance as it influences the drug release kinetics as well as the diffusion of various growth factors through permeable hydrogel surfaces.

**Fig. 1:**
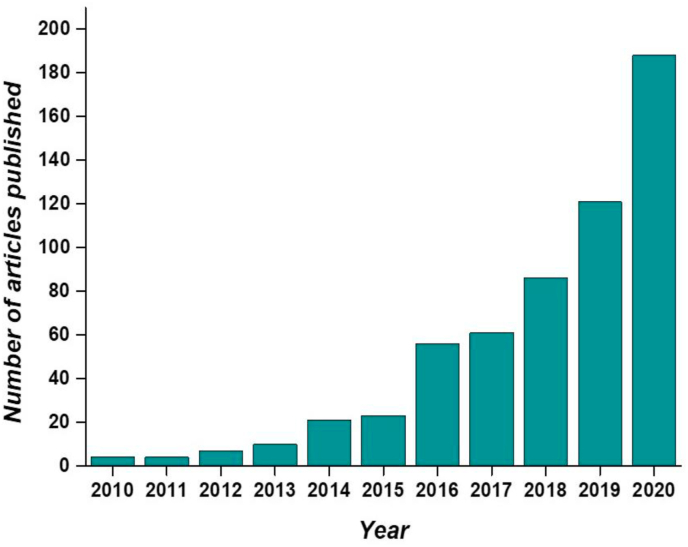
Number of articles published related to GelMA hydrogel during the last 10 years according to the ISI WOS (Web of Science) (report acquired on 16^th^ March 2021 using advanced keyword search; keyword- GelMA hydrogel).

In this review we emphasize multifunctional GelMA platforms with nanomaterials from different aspects, including its fabrication, crosslinking, polymeric–nanophase interactions, and its application as tissue therapeutics with future perspectives. The key objective is to provide critical analysis of multifunctional GelMA-nanomaterial platforms as versatile substrates and insights about their current challenges and future directions from a biomaterial point of view to their regenerative applications. Even though, many conceivable applications of these multi-functional materials are known, we focus more on its biomedical applications highlighting its current trends in regenerating tissues. (Fig. 1) shows the trend in GelMA related research during the last 10 years showing a substantial increase in the number of publications which marks the importance of these multi-responsive platforms.

## 2 Regulating the physicochemical properties: GelMA as a photocrosslinkable hydrogel

Hydrogel formation occurs by the cross-linking of polymer chains dispersed in an aqueous medium through numerous mechanisms, including physical gelation, ionic interactions, and chemical crosslinking [8,12,17,35,36,[54], [55], [56]]. Most of the physical gelation methods are temperature dependent and are reversible which depends on the inherent properties of polymers. In this case, the hydrogel can be obtained without further modification of polymer chains, but thermal reversibility is a typical drawback. The unsubstituted gelatin only forms physical crosslinking at specific concentrations and temperatures which results in hydrogels of inferior mechanical properties [55,[57], [58], [59], [60], [61]]. Among the numerous conceivable mechanisms for crosslinking proposed, chemical approaches are more precise and controllable in a spatially and dynamically distinct manner to recover the hydrogel stiffness [55,[61], [62], [63], [64]].

**Fig. 2:**
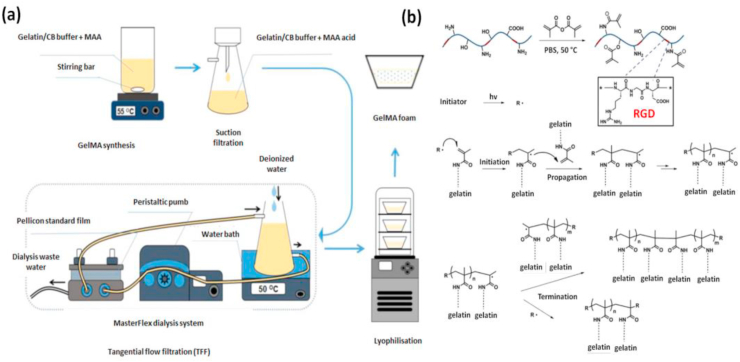
Representative images showing the synthesis and mechanism of crosslinking in GelMA hydrogel **(a)** Synthesis of GelMA hydrogel. Reproduced with permission from Ref. [38] Copyright © 2019 Nature Publishing Group. **(b)** The reaction mechanism indicating UV-induced free radical polymerization of GelMA hydrogel. Reproduced with permission from Ref. [37] copyright © 2015 Elsevier.

Due to the abundance of unsaturated photocrosslinkable groups present, substituted gelatin derivatives are highly prone to light-induced reactions. Primary amine (-NH_2_) and hydroxyl (-OH) groups are mainly involved in this substitution reaction where methacryloyl groups are introduced onto gelatin. The polymerization of GelMA occurs in an aqueous state by a free radical mechanism in presence of a photoinitiator. The UV irradiation of the photoinitiator causes the generation of free radicals by homolytic cleavage which initiates chain-growth polymerization. In the second step chain propagation occurs between methacryloyl groups present on the polymeric chain and finally terminates between a propagating chain and another free radical [[65], [66], [67], [68]]. (Fig. 2). Compared to other methods, photo polymerization displays numerous benefits, such as injectability, rapid gelation, improved mechanical properties, suitability for customized bioprinting along with easy incorporation with various cell types [67,[69], [70], [71], [72], [73], [74], [75], [76], [77], [78]]. However, the free radicals generated during crosslinking can attack cell membranes and results in cell death [67,69,75,77,79]. But, this effect depends on the source of radiation and intensity of UV light. Studies have also stated that photo crosslinking at milder conditions is biocompatible, which can be easily tuned by reducing the intensity from UV source and amount of photoinitiator used. It has also been reported that a high density of methacryloyl groups can have protective effects for incorporated cells [80,81].

**Fig. 3:**
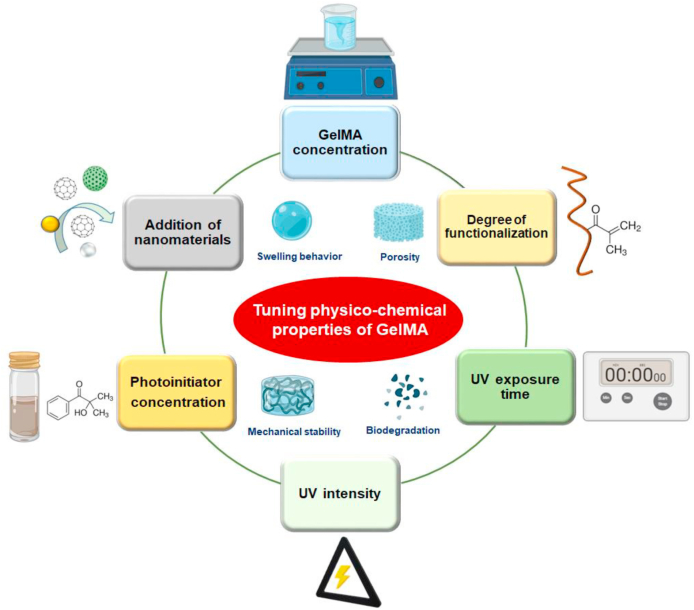
**(a)** Graphical summary of various factors that contributes to the physicochemical properties of GelMA. Tuning physicochemical properties are essential while engineering hydrogels as it contributes to better biological properties of the material in response to cells.

Based on former studies, it was observed that many factors influence the crosslinking kinetics and thereby the mechanical stiffness of photopolymerized GelMA which is a crucial factor that governs stem cell fate determination (Fig. 3). The mechanical properties of GelMA such as elasticity, compressibility, and hardness can be tuned by altering the prepolymer concentration, amount of photoinitiator used, photocrosslinking conditions, or by introducing many nanomaterials into the hydrogel matrix [[82], [83], [84], [85], [86], [87]].

### 2.1 GelMA concentration

Previous reports have shown the significance of GelMA concentration in influencing the physicochemical properties of resulting hydrogel and thereby its interaction on different cell types [37,38,88]. Studies revealed that when the GelMA concentration was increased, the elastic modulus of GelMA hydrogel also increased gradually along with its adhesive strength. Wu et al. reported the impact of the mechanical properties of GelMA substrate on the outgrowth of PC12 neural cell lines [89]. They investigated the influence of GelMA substrate stiffness on the cellular outgrowth using a range of hydrogels (5–30%) of varying stiffness. The 5% GelMA were found very soft compared to 10% GelMA which showed a young's modulus value ten times higher than 5% GelMA.

Dynamic shear oscillation studies conducted on low and highly methacrylated GelMA samples at various final concentrations (5%, 7.5%, and 10% w/v) showed a higher magnitude of storage modulus (G^I^) than loss modulus (G^II^) for both types of GelMA at all concentrations tested. When the GelMA concentration was increased from low (5%) to high (10%) at the same methacrylation rates, the G^I^ and G^II^ values were also increased. At the same time, it was also reported that GelMA samples analyzed at equal final concentration showed analogous G^I^ values, which was confirmed for both gelatin degrees of substitution. Moreover, it was confirmed that the temperature does not significantly contribute towards the hydrogel mechanical stiffness as evaluated by the comparable outcomes attained for all samples tested at 25°C and 37°C.

### 2.2 Degree of functionalization

It is known that higher substitution rates in GelMA could lead to a higher density of crosslinking sites that further influence the visco-elasticity of the resulting GelMA network [37,53,90,91]. The extent of substitution could affect the properties such as porosity, mechanical stiffness, and swelling behavior of the hydrogel. Therefore, many synthetic methods to enhance the degree of substitution were proposed, such as by using polar aprotic organosulfur compounds like dimethyl sulfoxide (DMSO) as the solvent for reducing the interaction between methacrylic anhydride (MA) and water as proposed by Martineau and co-workers [92]. Lee et al. suggested the idea of replacing phosphate buffer (PB) with carbonate-bicarbonate (CB) buffer system to increase the substitution rates in GelMA during the synthesis [93]. Later, Nichol and co-workers studied the role of degree of methacrylation on the mechanical features of resultant GelMA hydrogels by unconfined compression tests for low, medium, and highly substituted samples of varying concentrations (5%, 10%, and 15%) [88]. Collectively, the mechanical properties for the three sets of hydrogels enhanced at all strain levels analyzed owing to the higher degrees of substitution. The compressive modulus of GelMA reported at varying degrees of methacrylation was significantly higher at both 10% and 15% concentrations. This performance was unswerving at 5% GelMA, yet the change was not statistically significant.

### 2.3 UV crosslinking

Being a photocurable hydrogel, GelMA and its properties could be deployed in a Spatio-temporal manner by exposing it to UV radiation to accomplish the desired functionality [68,71,[94], [95], [96], [97]]. Among the various factors affecting the mechanical properties of GelMA, the parameters related to the source radiation such as its intensity, exposure time, and photoinitiator concentration are considered as utmost external control factors to manipulate the properties of hydrogels [54,[98], [99], [100], [101], [102], [103]]. The mechanical properties of GelMA hydrogels are easily influenced by the type and concentration of photoinitiators used. Free radical photoinitiators like 2-hydroxy-40-(2-hydroxyethoxy)-2-methylpropiophenone(IC-2959) and lithiumphenyl-2,4,6-trimethylbenzoyl phosphinate (LAP) are the most commonly used ones owed to their excellent biocompatibility and minimal immunogenicity [55,[104], [105], [106], [107], [108]]. Studies on the efficiency of photocrosslinking reactions using various concentrations of LAP by in-situ photo-rheology indicate a direct relationship of G^I^ with the degree of crosslinking. All analyzed samples showed a time-dependent increase in G^I^ though the percentage of crosslinking increases with increasing concentration of LAP. Connell and co-workers also confirmed the influence of UV intensity on the mechanical properties of GelMA by in-situ photo-rheology [97]. Measurements displayed an increase in G^I^ measured for a series of UV intensities. Even though altering the UV intensity had a noticeable effect on crosslinking kinetics, it does not significantly improve the final G^I^ of the hydrogels.

Mechanical compression tests on GelMA and hyaluronic acid methacrylate composite (GelMA/HAMA) hydrogels as a function of the duration of UV exposure at high intensity using LAP, IC2959, and 2,2′-azobis [2-methyl-*N*-(2-hydroxyethyl) propionamide (VA086), an azo initiator of same concentration showed variation in Young's modulus among hydrogel groups [109]. Among the three initiators analyzed VA086 resulted in hydrogels of lower moduli and LAP resulted in gel with a higher modulus. At a shorter crosslinking time both IC-2959 and VA086 failed to form stable hydrogels but at the same time LAP crosslinked and resulted in stable-stiffer hydrogels. IC2959 did not form stable hydrogels at shorter UV exposure due to the interference of photopolymerization reaction by permeable molecular oxygen (O_2_) [110,111]. Studies on the efficiency of photo-crosslinking reactions using various concentrations of LAP by situ photo-rheology also indicate a direct relationship of G^I^ with the degree of crosslinking. All analyzed samples showed a time-dependent increase in G^I^ though the percentage of crosslinking increases with increasing concentration of LAP. After approximately 60 s of UV exposure, 0.1% LAP attained its maximum G^I^, while 0.05% LAP took more than 250 s to accomplish its maximum G^I^. These results infer the benefits of incorporating a higher concentration of photoinitiators during UV crosslinking [112,113]. Remarkably the influence of dissimilar photoinitiators and crosslinking time on the mechanical properties of GelMA/HAMA hydrogel analyzed by compressive tests thus indicates that the compressive moduli of hydrogels could be enhanced by controlling the duration of UV crosslinking. Noshadi and coworkers used Triethanolamine (TEA), amphiphilic *N*-vinylcaprolactam (VC) a co-monomer, and Eosin-Y (EY) photoinitiators for their study [95]. It was observed that depending upon the VC, TEA, and EY concentrations used, GelMA hydrogels displayed variable compressive and tensile moduli. The compressive moduli of GelMA hydrogels showed a decrease in magnitude when VC concentrations were lowered at constant TEA and EY concentration. In addition to this, the tensile modulus of the hydrogels decreased gradually when the VC concentrations were dropped from higher to lower concentration at a constant TEA and EY concentration. Increasing VC and TEA concentrations would influence the reaction kinetics and the number of available crosslinking sites might increase at constant UV exposure which can lead to an enhanced stiffness.

## 3 Nanomaterials used for bio-functional GelMA systems

**Fig. 4:**
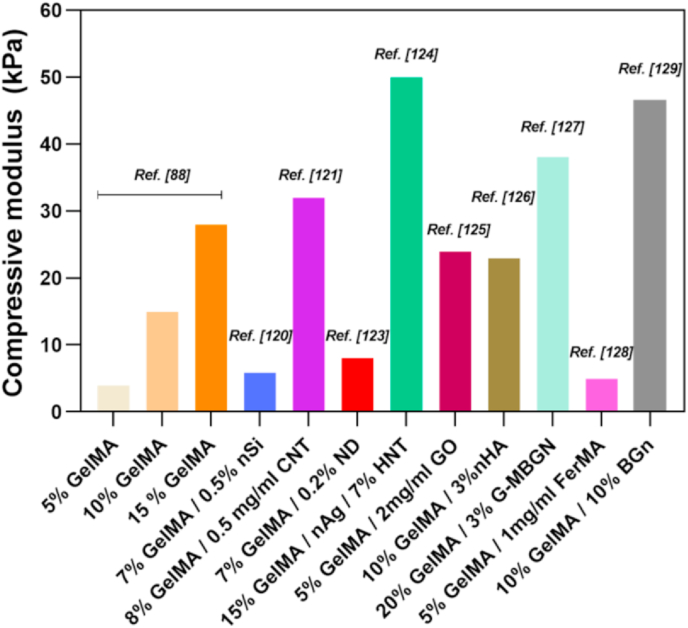
Bar diagram showing a trend in mechanical properties of GelMA platforms after incorporating various nanomaterials [88,120,121,[123], [124], [125], [126], [127], [128], [129]].

Like conventional hydrogel systems, the major drawback of pure GelMA is that it holds fragile properties that restrict its usage for specific tissue engineering applications [37,80,90]. The incorporation of various nanomaterials within GelMA hydrogel has thus evolved as a convenient way for developing multifunctional hydrogels of tailored functionality. Interposing a nano-architecture into GelMA appreciably influences its overall mechanical and biological properties. Both physical and covalent integration of nanomaterial into GelMA showed better mechanical properties in its nanostructured hydrogels which could be ascribed to their unique networking and proper entrapping of nanomaterials [37]. Such trends in enhanced mechanical properties of GelMA after nanomaterial addition are summarized in (Fig. 4). Apart from acting as stiffness-enhancing components, in certain cases, nanomaterials just behaved as physical fillers in the hydrogel matrix and were not chemically bound to the resultant hydrogels [24,114]. The conjugation between polymer and nanomaterials allows the transfer of mechanical force within the crosslinked networks ensuring higher mechanical strength and toughness [24,[115], [116], [117], [118]]. These modified hydrogels also own advantages such as supports excellent cell viability, differentiation ability, and formation of a stable vasculature [22,26,49,87,119,120]. Studies have revealed that multifunctional GelMA platforms with nanomaterials can tolerate high degrees of deformation such as compression, bending, elongation, tearing and shown to exhibit an increase of both G^I^ and Young's modulus as compared to pure GelMA of the same concentration. In a study, Cha and co-workers reported that graphene-based nanomaterials have an imperative role in strengthening GelMA hydrogels [87]. They demonstrated that incorporation of graphene oxide (GO) had a greater influence on the toughness than the rigidity of resulting hydrogels irrespective of its mode of incorporation. Furthermore, the addition of carbon nanotubes (CNTs) into the GelMA also leads to a higher modulus, while negligibly affecting the ultimate stress of the hydrogel structure [121]. Studies by Navaei et al. also indicate the same trend after the incorporation of gold nanorods (GNR) into GelMA [122]. They predicted hybrid hydrogels could resist deformation under force owing to the improved structural integrity of the matrix brought about by the electrostatic interaction between GelMA and GNRs, in fact, they act as reinforcing agents that improve mechanical properties.

**Fig. 5:**
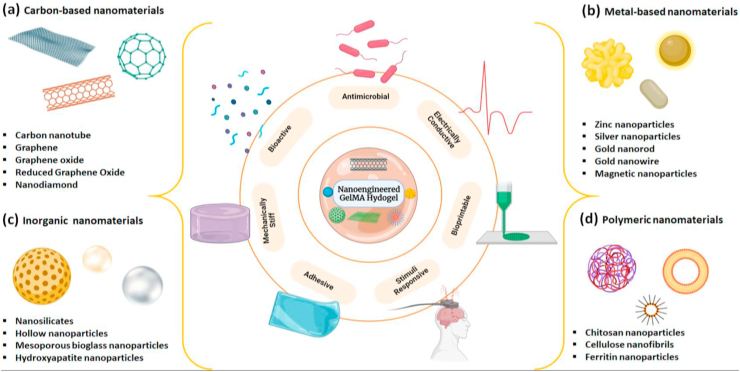
Summary of various nanomaterials used for engineering bio-functional GelMA hydrogels **a)** carbon-based **b)** metallic **c)** inorganic and **d)** polymeric nanomaterials. By tuning the hydrogel-nanomaterial interactions these multi-functional systems are used for various tissue engineering applications.

Apart from its role in improving mechanical features, the micro and nanoscaled features provided by nano-engineered GelMA have inspired in designing various smart hybrid hydrogels exhibiting stimuli-responsiveness. These advanced systems are required for various biomedical applications ranging from on-demand drug delivery to stimuli-driven tissue regeneration. Most of the smart platforms consist of carbon and metal-based nanomaterials due to their innate electrical and magnetic properties. Through advanced synthetic and fabrication techniques, it became so easy to tune these hybrid systems according to one's desire for specific applications such as soft bioactuation, biorobotics, biosensing, etc. [130,131] Jalili and his colleagues reported a stimuli-responsive injectable hydrogel formulation based on a thermoresponsive polymer and nanoengineered GelMA. The hybrid gels showed excellent magnetic and temperature-dependent release of doxorubicin (DOX) [132]. Due to these features, it was suggested for demand-based drug delivery. Another recommended hybrid system was based on vertically aligned CNT and GelMA used for smooth muscle regeneration [133]. This nano-engineered hydrogel exhibited anisotropic electrical conduction and biophysical features as compared to non-engineered GelMA. Studies also confirmed the role of CNT alignment in myogenic gene expressions after a set of experiments using electrical stimulation. Similar kinds of the study were done by many researchers using the same components such as Ahadian et al. [134] and Shin et al. [135] The CNT incorporated bio-hybrid actuators developed by Shin's group were noted for their unique impulsive actuation, tunable conductivity, and mechanical integrity. This smart-biomaterial showed excellent results with in-vitro cardiac cells, gaining special interest in designing stimuli-responsive biomedical devices. The following section summarizes various bio-functional GelMA hydrogels resulted from diverse types of nanomaterials (Fig. 5) and their properties intended for specific regenerative applications are summarized later in (Table 1).

**Table 1:** Summary of multifunctional GelMA platforms with nanomaterials exploited for various tissue engineering applications.

Type	Component	Properties of composites	Target application	Reference
Carbon-based	CNT	Reinforcing GelMA and improved electrical conductivity	Cardiac tissue engineering	[146]
	CNT	Cell-responsive, stiffness dependent hydrogel platform	Cell encapsulation	[121]
	GO	Tunable physical properties and enhanced cellular behavior	Cell encapsulation	[125]
	GO	Gene delivery approach by promoting myocardial vasculogenesis	Acute myocardial therapy	[260]
	GO	Biocompatible photopolymerizable bio-ink for promoting chondrogenic differentiation	Cartilage tissue engineering	[263]
	rGO	Better mechanical property, electrical conductivity, and improved cardiac function	Cardiac tissue engineering	[154]
	rGO	Enhanced electrical conductivity and biocompatibility	Nerve tissue regeneration	[264]
	rGO	Better mechanical properties, electrical conductivity, flexibility as well as permeability	Peripheral nerve regeneration	[265]
	ND	Enhance the stiffness of GelMA matrix, drug loading, and sustained release	Bone regeneration	[123]
Inorganic	nSi	Improved mechanical properties, osteogenesis ability in absence of osteoinductive agents	Bone tissue engineering	[266]
	2D-nSi	ECM mimicking scaffolds, Osteogenic differentiation in growth factor free environment	Bone tissue engineering	[120]
	BGN	Improved bioactivity, biocompatibility and enhances bone formation	Bone tissue engineering	[129]
	nHA	3D printed biomimetic multilayered scaffolds	Osteochondral regeneration	[185]
Metallic	Au-NP	Improve mechanical properties and promote osteogenesis	Bone tissue engineering	[267]
	Au-NR	Electrically and mechanically enhanced material Characteristics	Cardiac tissue engineering	[122]
	Au-NW	Improved electrical conductivity and mechanical properties	Cardiac tissue engineering	[216]
	MNP	Mechanically stiff nanocomposite hydrogel at ultralow nanoparticle content	Scaffolding biomaterial	[218]
	AgNP	Accelerated wound healing, antibacterial effects	Skin tissue engineering	[217]
Polymeric	CNF	Low concentration bio-ink for 3D bioprinting	Skin tissue engineering	[268]
	n-Chitosan	Sustained release of angiogenic growth factor	Blood vessel formation	[259]

### 3.1 Carbon-based nanomaterials: stiffness and conductivity

Among the various regenerative applications offered by nanomaterials, conjugated and modified carbon-based nanomaterials are in the frontline used for the advancement of nano-biomaterials that contributes positive outcomes in numerous therapies and to overcome drawbacks in preclinical applications. Nanomaterials like graphene, GO, CNT, reduced graphene oxide (rGO), and nanodiamonds (ND), etc. have exposed potential applications towards regenerating tissues [46,[136], [137], [138], [139], [140], [141], [142]]. These materials retain remarkable electrical, mechanical, and optical properties due to their characteristic nano-structure and strength of the bonds between carbon atoms. But the presence of hydrophobic groups on the periphery restricts their interaction with hydrophilic polymers. To overcome these shortcomings nanomaterial surfaces are modified with various polar functional groups such as –OH, –NH_2_, and carboxyl (-COOH) or enhancing their dispersion by grafting it with different polymer chains [46,[143], [144], [145]]. Due to the presence of long carbon chains and prevailing sp^2^ hybridization CNTs act as reinforcing agents within the GelMA network and make the hydrogel responsive to external electrical or thermal stimuli. These hydrogels are widely used to engineer different electroactive tissues such as neurons, cardiac and muscular tissues due to their improved electrical conductivity. Shin group succeeded in reinforcing GelMA hydrogels using CNTs functionalized with multiwalled –COOH groups offering a fibrous structure within the interconnected and spongy hydrogel network where CNTs are also present [146]. An addition of 0.5% of modified CNTs into the hydrogel enhanced its tensile modulus to triple fold due to the formation of a nanofibrous mesh-like network of GelMA coated with multiwalled CNTs. Studies demonstrated that the cardiomyocytes seeded onto two-dimensional CNT/GelMA nanocomposite surfaces enhanced the spontaneous beating frequency in multiple folds as compared to the non-engineered hydrogel groups.

**Fig. 6:**
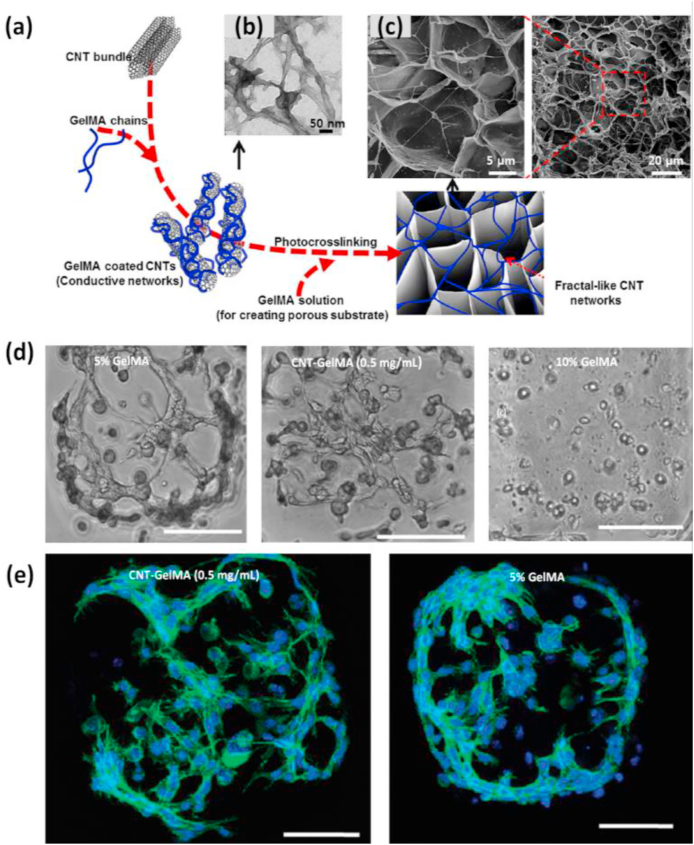
**(a)** Graphics indicating synthesis of complex CNT networks encapsulated inside GelMA hydrogel. **(b)** TEM images of GelMA-coated on CNTs. **(c)** SEM images showing thin CNT/GelMA hybrid film (1 mg/ml) showing its porous surface morphology. The zoomed view shows fiber-like structures across and inside the porous network. Reproduced with permission from Ref. [146] copyright © 2013 American Chemical Society. **(d)** Images showing cell distribution in 5% GelMA, CNT/GelMA (0.5 mg/ml), and 10% GelMA after 48 h of culture. **(e)** Confocal phalloidin/DAPI images of cells showing the formation of interconnected networks in micropatterned CNT/GelMA hybrid platform and 5% GelMA after 48 h of culture. Reproduced with permission from Ref. [121] copyright © 2012 American Chemical Society.

A similar study showed that CNT-reinforced GelMA hybrid hydrogels also act as a cell-compatible, cell-responsive hydrogel platform for generating cell-laden three-dimensional (3D) constructs [121]. CNTs were coated with a thin layer of GelMA by utilizing hydrophobic interactions arising between the polypeptide chains in the GelMA backbone and the sidewalls of CNTs without considerably affecting its physical properties. The thin layer coating enabled uniform dispersal of CNT in the GelMA solution and also enhanced the mechanical properties due to the formation of a nanofiber web-like structure that resulted in covalent bonding between GelMA and CNT (Fig. 6). Besides maintaining the mechanical properties of hybrid hydrogels, the nanofibrous units formed after the addition of CNTs could also conserve the beneficial bioactive properties of the GelMA such as the inclusion of bioactivity, highly porous morphology, and biodegradability. The CNT/GelMA hybrid platforms showed promising results with human mesenchymal stem cells (hMSCs) and seemed to modulate the cell morphology. Due to the cell supportive features, these composites show promising applications in creating artificial 3D cellular microenvironment mimicking scaffolds to guide stem cell differentiation to neurons, muscles, bone, cartilage, cells in a stiffness-dependent manner [134,[147], [148], [149]]. Moreover, it can also be used for in-vitro cell studies or fabricating complex 3D biomimetic tissue-like structures due to the high pattern fidelity and resolution of CNT/GelMA hybrid hydrogels.

The cellular response initiated by carbon-based nanomaterials was also evident from a recent study conducted on a 3D culture platform made by incorporating GO homogenously into the GelMA hydrogel [125]. GO contains oxygenated hydrophilic groups which reduce the irreversible agglomeration of GO sheets through π-π stacking and van der Waals interactions [[150], [151], [152], [153]]. GO/GelMA hydrogel system with tunable physical properties thus enhance cellular behavior and could be used as a microscale tissue-engineered scaffolding material. The addition of rGO into the GelMA hydrogel also considerably enhanced its electrical conductivity and mechanical features [154]. Additionally when cultured with cells a novel rGO/GelMA platform displayed better cell survival, proliferation, and growth as compared to the cells cultured on GelMA only. The contractile behavior and spontaneous beating rate of cardiomyocytes improved significantly after culturing them on rGO/GelMA hydrogel sheets as compared to those cultured on pristine GelMA of similar mechanical properties. The strategy of incorporating rGO within GelMA thus paved the way to engineer high-fidelity tissue models for clinical applications and the in-vitro regeneration of cardiac tissues.

The interest in carbon-based NDs for biological and medical applications are also on the rise over recent years. These materials offer benefits for moderating the physical and biological properties of ND-based theranostic platforms. Even though the diamond core is chemically inert, a large number of functional groups present on its surface can be conjugated for suitable tissue engineering applications [[155], [156], [157], [158], [159], [160]]. Several synthetic techniques have led to the design of modified NDs by functionalizing them using –NH_2_, –COOH, or fluorescent groups. The easily tunable functional groups on NDs have allowed scientists to validate the delivery of proteins and nucleic acids for therapeutics [155,[161], [162], [163], [164], [165], [166], [167]]. Apart from their drug delivery nature, less cytotoxicity also makes them versatile among other carbon-based materials such as graphene and CNTs. Studies using ND incorporated GelMA revealed that they simultaneously act as nano-filler and carter of corticosteroid dexamethasone (DEX), to influence the stiffness and osteogenic differentiation potential of GelMA hydrogels [123]. Studies on the compressive modulus and shear rheology of hydrogels of varying stiffness indicate that NDs have a key role in enhancing the mechanical properties of GelMA hydrogels. Indirect evaluation of the traction forces on human adipose-derived stem cells (hASCs) also confirms the same. Besides these ND/GelMA platforms exhibited sustained release of DEX compared to GelMA due to the active adsorption of DEX on the ND surface. Finally, hASCs encapsulated within the ND-DEX complex promoted its differentiation towards osteogenic lineage. The results indicate the benefits of ND incorporation in the design of gelatin-based nano-biomaterials and their application in the handling of non-load-bearing defects of bone tissues.

**Fig. 7:**
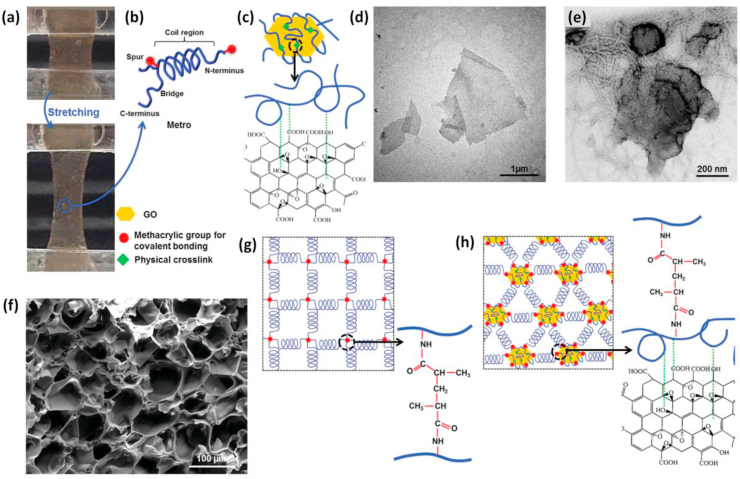
Fabrication of MeTro/GO/GelMA hybrid platforms. **(a)** Demonstrative pictures showing highly elastic MeTro/GO made using GO (1 mg/ml) and MeTro prepolymer solution (10%) before and after the stretching. **(b)** Shows MeTro molecules with an irregular coil and a C-terminal cell interactive groups **(c)** Shows the binding of GO with MeTro utilizing hydrophobic interactions. The figure also shows representative **(d)** HRTEM images of dispersed GO and **(e)** MeTro bound GO particles, representing tropoelastin ﬁber covering over GO particles. **(f)** SEM images of highly interconnected MeTro/GO hydrogel network **(g)** Representation of MeTro hydrogel network formed by photopolymerization. **(h)** Formation of covalently conjugated MeTro/GO hydrogel employing UV irradiation and physical interaction between GO particles and MeTro molecules. Reproduced with permission from Ref. [173] Copyright © 2016 John Wiley and Sons.

Recent approaches also focussed on using biomimetic elastomers, which are among the key components of ECM of elastic tissues for engineering damaged tissues by conserving their distinctive bioactivity [[168], [169], [170], [171], [172]]. Methacryloyl-substituted tropoelastin (MeTro) and GO hybrid hydrogel, a conductive and elastomeric biocompatible material based on GO nanoparticles and recombinant human tropoelastin protein developed by Annabi and co-workers is an example [173]. The key aim of this study was to disperse GO nanoparticles unvaryingly throughout the MeTro solution to generate physical crosslinking which offers elasticity to the GelMA matrix while preserving the protein coil region. MeTro/GO hybrid hydrogels have better elasticity and toughness as compared to the other existing hybrid platforms due to the exceptional hydrophobic and electrostatic interactions between tropoelastin polymer chains and GO (Fig. 7). In short, the engineered GelMA owns tunable mechanical, electrical, and biological properties due to their unique composition and physical structure. These properties and cell supportive features make them highly demanded platforms for various applications including the development of cardiac tissue constructs, actuators, and bio-electronic devices.

### 3.2 Inorganic nanomaterials: reinforcement, therapeutic ions, and bioactivity

**Fig. 8:**
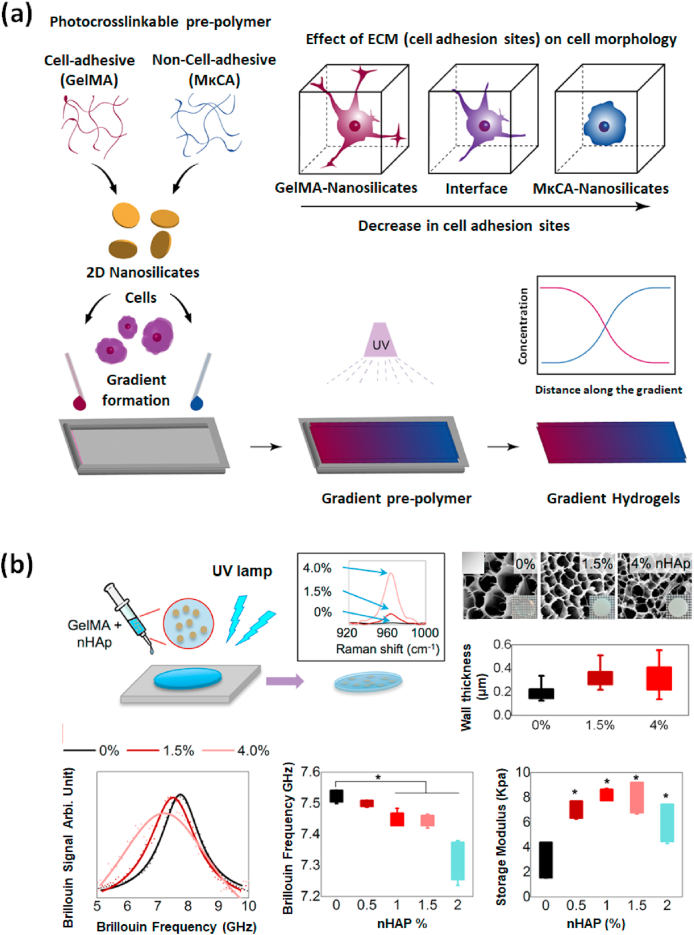
**(a)** Gradient hybrid platforms composed of GelMA and 2d-nSi formed by UV crosslinking. The presence of RGD groups in GelMA supports cell attachment, spreading while MκCA which lacks cell-binding sites fail to spread and maintain morphology over time. Reproduced with permission from Ref. [188] Copyright © 2018 Elsevier. **(b)** Fabrication and probing the mechanical properties of nHAp/GelMA hybrid platform using Brillouin spectroscopy. Results show the relationship between the Brillouin spectroscopy outcomes and the attained rheology data. Reproduced with permission from Ref. [83] copyright © 2017 American Chemical Society.

Inorganic nanomaterials have emerged as the most valued functional building blocks widely used for engineering tissues and delivery purposes [49,[174], [175], [176], [177], [178], [179], [180], [181]]. Many distinctive properties of inorganic nanomaterials are particularly useful in the design of nano-biomaterials. The protein adsorption and corona formation by inorganic nanomaterials entrapped inside the hydrogel is an intricate process. It provides a vital basis for the attachment of cells where the pure hydrogel formulation often lacks the mandatory cell-binding sites like RGD [[182], [183], [184]]. But GelMA lacks such drawbacks due to the richness of many RGD-motifs which supports cellular adhesion by sensing the integrin receptors present on the surface of most cell lines. In recent years, a wide variety of bioactive inorganic nanomaterials incorporated within GelMA has been reported including nanohydroxyapatite (nHAP) [[185], [186], [187]], nanosilicates (nSi) [120,[188], [189], [190]], bioactive glasses (BGn) [129,[191], [192], [193]], mesoporous silica (MSN), etc. [194,195] Most of these inorganic nanoparticles support cellular functioning and are indispensable for the normal activities of human tissues. Paul et al. reported that nSi-based biocompatible hybrid GelMA platforms can induce osteogenic differentiation of the encapsulated hMSCs even in absence of osteoinductive agents like bone morphogenic protein (BMP), a multifunctional growth factor, or DEX, an osteoinductive factor [120]. The potential applications of nSi/GelMA hybrid hydrogels are inspected using encapsulated hMSCs for the determination of production of ROS, apoptotic caspase activities, and inflammatory responses. Studies on gradient hydrogel systems formed by disc-like two-dimensional (2D)-nSi also showed their role in controlling the physicochemical and biological properties of resultant hybrid hydrogels. Comparative studies on gradient-controlled GelMA and methacryl functionalized kappa carrageenan (MκCA) incorporated with 2D-nSi using a micro-engineered flow channel showed characteristic properties in hybrid platforms. The improved shear-thinning, mechanical, and cell adhesive properties on 2D-nSi/GelMA hydrogels are attributed to the stiffness regulation by 2D-nSi and cell supportive features of GelMA [188] (Fig. 8a). Such gradient hydrogel system could be applied for tissue interface regeneration where consistent Spatio-temporal changes exist.

Studies conducted on the mechanical stiffness of GelMA-nanomaterial platform made by the incorporation of nHAP, using dual Brillouin/Raman spectroscopy showed a non-uniform distribution of mechanical stiffness at micrometer range (Fig. 8b) [83]. Moreover, the chemical information associated with the hydrogel samples was revealed without extra operations. Extensive studies are still going on in enquiring about the mechano-chemical features of nanostructured hydrogels using these advanced non-contact and non-invasive techniques [[196], [197], [198]]. The high surface reactivity of inorganic nanomaterials is one of the crucial factors to be considered while designing bioactive composites for bone tissue regeneration [181]. The biological interface resulted from the contact of inorganic ions with physiological fluids generates strong bonds formed within the bone tissues. Similarly, the negatively charged oxygen surface of inorganic ceramics could chemically conjugate with the positively charged –NH_2_ functional groups of hydrogels utilizing electrostatic interactions [[117], [199], [200], [201]]. Polar amino acids like lysine, hydroxylysine, histidine, and arginine can actively participate in this type of reaction due to their innate positive charge. Alternately secondary interactions could also occur either by hydrogen bond formation between –OH and carbonyl groups (–C=O) or by electrostatic attractions that have the potential to attract organic groups [17,22,24]. BGn-based biomaterials are also striking platforms for repairing damaged bones due to good structural integration with the surrounding bone tissues as well as their capacity for enhanced bone formation. [[202], [203], [204], [205], [206]]. Other inorganic nanomaterial-based platforms are only photocrosslinkable while biomimetic BGn/GelMA composite hydrogels were fabricated through a dual crosslinking approach (physical gelation + UV-based chemical crosslinking) [129]. The BGn/GelMA hybrid hydrogels showed improved bioactivity and stability. Moreover, the photocrosslinking process avoided the use of toxic crosslinkers which is a typical drawback of photopolymerized systems. Novel photocrosslinkable inorganic strengthened hydrogel membrane embedded with mesoporous bioactive glass nanoparticles (MBGNs) reported by Xin and colleagues were noted for its angiogenesis and osteogenesis potential [127]. This hybrid-biomaterial in form of a membrane exhibited better mechanical features, resilient degradation behavior, long-term ion release, stability at a wide range of pH, and displayed mineralization of tissues. The accelerated new lamellar bone formation in rat calvarial defects specified its promising application in in-vivo systems. This composite system has been successfully used for the development of biomaterials that can act as artificial periosteum with high regenerative properties. The basic idea behind developing inorganic nanomaterial-based GelMA platforms is to improve the mechanical properties and bioactivity of GelMA. The chemical interactions existing between the inorganic nanomaterial phase and the GelMA matrix can allow different interfacial properties offering mechanical improvement. This can also alter the degradation behavior of the nano-engineered hydrogels allowing them to find various applications in soft and hard tissue engineering.

### 3.3 Metallic nanomaterials: stimuli-responsiveness

The hydrogels integrated with metallic nanomaterials have become an evolving area in developing customized multi-responsive constructs for tissue repair. Nano-biomaterials based on metals and their oxides have been shown to possess desired physical properties such as conductivity of electricity (gold-based) [207], magnetic behavior (iron-based) [208], and antimicrobial action (silver-based) [209]. Thus hydrogels engineered with metal or metal-based nanomaterials have extensive applications and are actively used as drug delivery systems, conductive scaffolds, bioelectronic units, bio-imaging, and sensing agents [[210], [211], [212], [213], [214]]. The existence of weak interactions between the hydrogel and the metallic nanomaterials can limit its applications. However, modifying the surface chemistry of nanomaterials could exert interactions among the hydrogel and nanophase and considerably influences the physicochemical, and biological properties of resulting hybrid systems. The role of gold nanoparticles (GNPs) for engineering defected bones was investigated using GelMA. GNPs displayed a progressive effect on the differentiation of mesenchymal stem cells (MSCs) and MC3T3-E1 osteoblast-like cells to osteogenic lineage [215]. GNP-laden hydrogels supported maturation, spreading, differentiation, and alkaline phosphatase (ALP) activities of adipose-derived stem cells (ADSCs) as they differentiate towards osteogenic lineage in a dosage-dependent manner. Efficiency of in-vivo bone formation was assured by these platforms at a higher concentration of GNPs.

**Fig. 9:**
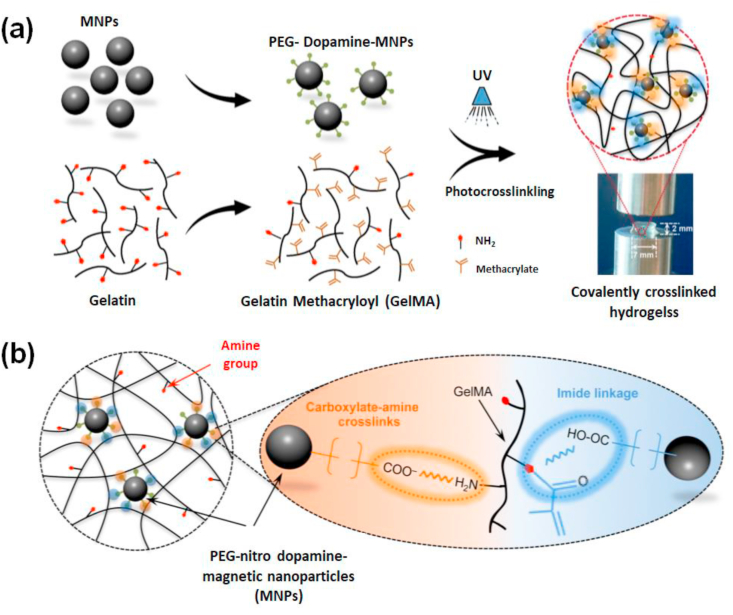
Graphics showing the formation of covalently crosslinked hydrogels by incorporating MNPs. **(a)** Photopolymerization results in mechanically stiff hydrogel platforms even at ultralow content of modified nanoparticles which act as crosslink epicenter **(b)** Illustrating chemical interactions between surface-modified magnetic nanoparticles with GelMA backbone. The free –NH_2_ functional groups on GelMA binds covalently with COO^−^ groups from functionalized nanoparticles and form stable amide crosslinking. Reproduced with permission from Ref. [218] copyright © 2016 American Chemical Society.

Navaei and colleagues reported a photopolymerizable hybrid platform based on GNR encapsulated within GelMA hydrogels [122]. This nanocomposite system unveiled good biological properties and was applied for developing functional cardiac patches. The GNR addition maintained the electrical conductivity and mechanical features of the GelMA matrix. Appropriate accommodation of cardiac cells on the nanocomposite system then promoted cell retention, proliferation, and most importantly uniform distribution of different cardiac-specific markers, cell-cell interactions, and harmonized tissue level beating behavior. Studies using water dispersed gold nanowire (GNW) of high-aspect-ratio incorporated within GelMA hydrogels also exhibited similar actions towards cardiac tissue engineering, making gold-based biomaterials one of the most favorable biomaterial scaffolds to regenerate cardiac tissues [216]. Lately, Jahan et al. used the antibacterial properties of silver nanoparticles (AgNP) incorporated with GelMA and quantified the motility of fibroblasts on this substrate [217]. The effectiveness of adding AgNPs to soft GelMA hydrogel aimed at faster wound healing of deep skin wounds. In another work, Jaiswal and co-workers succeeded in developing a mechanically stiffer nano-engineered hydrogel at extremely low amounts of metallic nanoparticles [218]. The improved mechanical properties could be credited to the chemical bonding between the gelatin backbone and surface-modified magnetic nanoparticles (MNP). Being photoreactive, nitro-dopamine conjugated to the surface of MNPs might act as a cross-link epicenter (Fig. 9). Compared to other stiffer GelMA hydrogels reported so far, the number of nanoparticles incorporated here was 2–3 times lower. A 10,000-fold lower concentration of nanoparticles compared to GelMA even contributed to a 10-fold increase in mechanical stiffness. GelMA/MNP also showed good cell viability when encapsulated with cells. Since all these metal-based nano-biomaterials respond to stimuli (both endogenous and exogenous), modern clinical therapeutics uses these smart scaffolds as candidates for cell encapsulation and for the selective release or localization of genes/drugs/proteins.

### 3.4 Polymeric nanomaterials: controlled delivery of bioactive molecules

Among the multitude of GelMA platforms developed for tissue engineering, polymeric nanomaterial incorporated ones have attained a remarkable interest. These structures are similar to the macromolecule-centered components in the human body and are often considered to be good candidates for drug delivery purposes since they offer spatial and sequential control over drug/gene/growth factor release owing to the tailored physical properties, manageable degradation as well as their capability to resist the degradation of labile biomolecules [142,[219], [220], [221]].

Such delivery systems can impact therapeutically advantageous properties of drug delivery and are set to possess clinical importance [[222], [223], [224], [225], [226], [227], [228], [229]]. Compared to microparticle or emulsion-based delivery systems hydrogel-based delivery systems are more effective since it retains protein stability during the synthesis [[230], [231], [232], [233]]. Most importantly the encapsulated protein molecules have restricted mobility or immobilization within the porous network, which is useful for the conservation of their well-defined 3D structure. The majority of the hydrogel-based reservoir systems are characterized by this property where the sustained release of proteins maintains an optimum concentration of proteins encompassing tissues and enhances its circulation time within the body over extended periods for achieving effective therapeutic results [[234], [235], [236]]. These properties of hydrogels are found to have a direct relationship with the crosslinking density [237], porous microstructure [238] as well as other existing chemistry [107]. Hydrogel binds with proteins through physical interactions governed by mechanisms such as diffusion, degradation, or even a concoction of these phenomena [[239], [240], [241], [242]]. Several other strategies are also known which monitor the controlled release of drugs from hydrogels such as using a secondary delivery system (nanoparticle or microparticles in composites) or relying upon reversible hydrogel-protein interactions.

Several polymeric nanomaterials such as hyperbranched polymers, dendrimers, nanopolysaccharides, proteins like ferritin, etc. have various hydrophobic or hydrophilic units available for conjugation with the functional groups of hydrogels [[243], [244], [245], [246]]. These properties encompass the application of polymeric nanomaterials for numerous delivery applications. Among these, dendrimer and hyperbranched polymer-based ones are more attractive candidates because of their highly branched chemical structure. Because of this inimitable nanostructure, they possess various active peripheral functional groups resulting in better reactivity and higher efficiency for loading bioactive molecules compared to their linear analogs [[247], [248], [249], [250], [251], [252], [253]]. A dual cross-linkable (physical and chemical) hydrogel platform based on GelMA and ferritin nanocage developed by Roya and co-workers is one such example [128]. Physical interactions arise between ferritin protein and its empty-core equivalent apoferritin with GelMA and the covalent conjugation by methacrylated ferritin and apoferritin resulted in hybrid nanocage embedded hydrogels which are fringed by the surface of the nanocage and GelMA matrix. Compared to the physically bonded ferritin and apoferritin made by the direct dispersion, chemically conjugated ones offered a better ability to tune the mechanical properties without altering the porous morphology or other cell supportive features. Moreover, cumulative release studies also revealed the potential of nanocage-based GelMA to release small molecules in response to pH stimuli. Previous studies also state that incorporating micro or nanocarriers into GelMA would facilitate the sustained release of growth factors [107,[254], [255], [256], [257], [258]]. Polymeric nanomaterial obtained from polysaccharide chitosan was successfully utilized for growth factor delivery attributed to its characteristic compatibility and degradation properties [259]. Nano-chitosan incorporated GelMA hydrogel comprising growth factors are designed to provide a sustained release of basic fibroblast growth factor (bFGF), an angiogenic growth factor that has a vital role in blood vessel formation. Followed by incorporation, the mass swelling ratio of hybrid hydrogel increased due to the hydrophilicity of nano chitosan which allows more water movement into the hydrogel matrix. Release studies indicate that chitosan nanoparticles loaded with bovine serum albumin (BSA) and bFGF show burst release especially during the first 2 h where 75% of the loaded protein was released. At the same time, GelMA/chitosan hybrid hydrogel loaded with BSA–bFGF sustained the release initially for 4 days (approx. 75%) and continued to more than 7 days (up to 90%). Cell supportive features such as proliferation analyzed for GelMA, GelMA/chitosan, and BSA– bFGF loaded GelMA/chitosan platforms indicates normal human dermal fibroblasts (NHDF) cells will survive and proliferate more on BSA– bFGF loaded GelMA/chitosan due to the release of growth factors into the culture medium. These promising in vitro results demonstrated the suitability of these hybrid nano-biomaterials for tissue engineering by growth factor delivery.

**Fig. 10:**
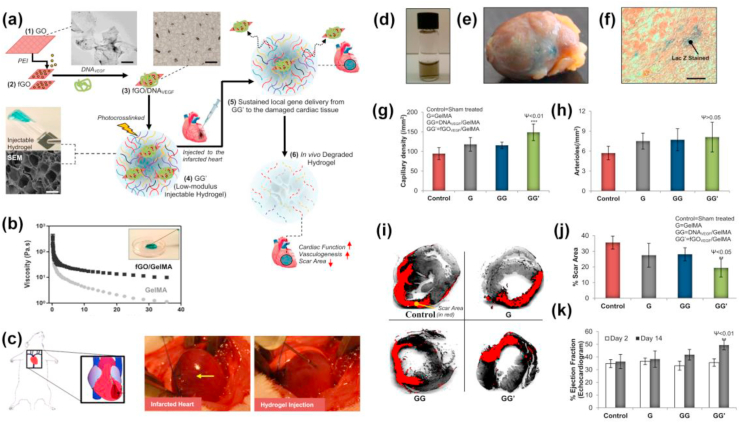
**(a)** Schematic steps of fabrication of injectable polymer functionalized GO/GelMA hybrid hydrogel platform for AMI therapy. **(b)** The injectability of GO/GelMA hybrid hydrogel as evaluated by shear rate analysis. **(c)** Graphics showing rat heart with AMI and real images showing infarcted rat heart and intramyocardial delivery for injectable fGO/GelMA nano-engineered hydrogel through the peri-infarct region respectively. **(d)** Photograph of bioactive hybrid hydrogel **(e)** X-Gal stained image of the explanted heart showing Lac Z gene expression **(f)** Histology images of ventricular tissue section at the microscopic level. Quantification of **(g)** capillaries and **(h)** arteriole densities in the peri-infarct region, after 14 days post-injection. **(i)** Illustrative images of the Sirius red-stained left ventricular myocardial section showing cardiac fibrosis regions in red and myocardium in grey. **(j)** Morphometric analysis of the left ventricle for determining scar area. **(k)** Cardiac function ECG indicates GO/GelMA showed better EF% than other groups. Reproduced with permission from Ref. [260] copyright © 2014 American Chemical Society.

A biocompatible injectable hydrogel gene delivery system developed by Paul and colleagues also showed its efficacy in specific delivery of vascular endothelial growth factor (VEGF), a signaling protein that supports angiogenesis that is necessary for the regeneration of damaged cardiac tissues for acute myocardial therapy (AMI) [260]. GelMA infused with cationic polymer functionalized GO nanosheets (GG^I^) acted as the delivery system for VEGF which efficiently transfect myocardial tissues which could facilitate local myocardial neovascularization at the injected sites, reduces fibrosis, and potentially improve cardiac function in an in-vivo AMI model as indicated in (Fig. 10). This nanoengineered hydrogel delivery system not only facilitated the sustainable release of angiogenic factors but also protected the delivered components from the harsh external environment within the beating heart. Such a 3D GO-based delivery system might be useful in directing cellular behavior in a 3D microenvironment and can be used for advanced tissue regenerative applications. Another GelMA based shear thinning and self-recoverable hydrogel platform was made by Jalili and co-workers by engineering its crosslinking density. They have developed thermoresponsive poly(*N*-isopropylacrylamide-co-acrylamide) nanogels loaded with DOX by entangling it within the GelMA prepolymer before photocrosslinking [132]. This nanoengineered hydrogel exhibited both temperature and flux-dependent release of DOX. The in-vitro DOX release studies on injectable hydrogels using preosteoblast and osteosarcoma revealed that these nanocomposite hydrogels may be used for on-demand and confined therapeutic delivery [118,261,262]. Recent researches on polymeric nanocarrier incorporated GelMA drew attention to its injectable form which can be administered for the minimally invasive surgery. Such delivery systems also show rapid gelation when the viscoelastic hydrogel prepolymer solutions are administered to the target site in response to change in temperature, pH, solvent type, or through crosslinking such as photopolymerization, coordination complex formation, or inclusion complexation, making them ideal for tissue repair.

## 4 Advanced GelMA-nanomaterial platforms for tissue therapeutics

**Fig. 11:**
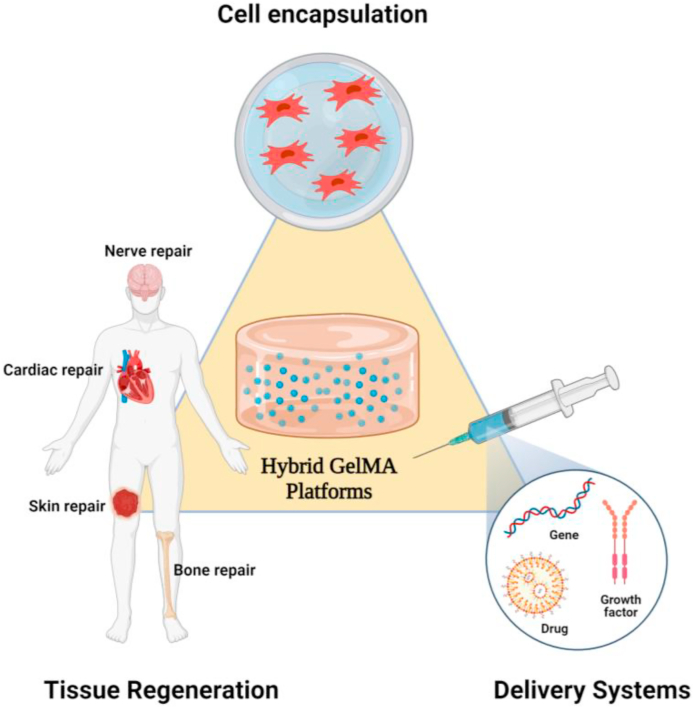
Systematic diagram showing various applications of engineered multifunctional GelMA platforms.

Tissue repair and reconstruction using nano-engineered hydrogels have been developed as a promising medical strategy to the existing replacement therapies for the healing of damaged tissues [22,26,[269], [270], [271]]. Tissue replacement possesses several drawbacks such as rejection by the immune system and prone to infections. Advanced tissue engineering techniques could be used to overcome these shortcomings in the field of regenerative medicine infections [[272], [273], [274], [275], [276]]. Fabricating novel hydrogel scaffolds or injectable formulation for stem cell therapy thus became frontiers in the modeling and treatment of various diseases [[277], [278], [279], [280], [281], [282]]. Such biomaterials capable of paralleling native tissues in-vitro conditions are later implanted in-vivo afterward to regenerate damaged tissue functionality [[283], [284], [285], [286], [287]]. These advanced platforms can mimic and simulate the organization and characteristics of the cellular microenvironment such as having a nanoporous morphology and functionality for cellular activities [22,26,288,289]. These properties make them one of the most versatile multi-responsive systems of boundless research interest. Researches during the last decade show the role of GelMA-nanomaterial hybrid platforms in engineering a wide range of tissues such as bone, cartilage, muscles, skin, cardiac, neural, and vascular tissues, etc. aside from its role in cell encapsulation and growth factor/drug/gene delivery. (Fig. 11) shows a schematic representation of various applications of engineered multifunctional GelMA-nanomaterial platforms.

### 4.1 Advanced platforms for bone tissue

Many new developments have been made in engineering bone tissues by using hydrogel as a multifunctional platform during the last decade. Even though hydrogel-based materials display excellent bio functionality it possesses demerits such as poor mechanical stability and low processability making it unsuitable for bone tissue regeneration [94,290,291]. Biomaterial design for bone tissue repair entails an understanding of the structure and composition of natural bone tissue, as well as choosing the apt biomaterials satisfying these requirements. Natural bone tissue possesses a structure that closely resembles nano-biomaterials that offers appropriate physical and biological properties [269,[292], [293], [294], [295], [296]].

Owing to many fascinating potential uses of multicomponent GelMA-based materials, their design and synthesis have been a focus of substantial research over the last few years**.** However, single-layered GelMA hydrogels often fail to regenerate multiple tissue defects such as osteochondral defects possibly due to the mismatched stiffness of the hydrogels relative to cartilage and subchondral bones. On the other hand, bilayered GelMA hydrogels based on mussel-inspired chemistry without nanomaterial engineering have been shown to restore such defects [255,297].

GelMA-nanomaterial interfaces provide an appropriate matrix environment and facilitate controlled growth factor delivery at different points of bone regeneration. In an early attempt studies using GNP encapsulated GelMA hydrogels (GNP/GelMA) revealed that the presence of GNP might promote cell spreading, osteogenic differentiation, and basic phosphatase activities of ADSCs [215]. In response to GNP/GelMA platform, ADSCs differentiated into osteoblast cells and consequently promoted new bone formation. Another novel hybrid system reported was based on nanosilver (nAg) and halloysite nanotubes (HNTs), which are tubular clay nanomaterials incorporated into GelMA (nAg/HNTs/GelMA) [124]. This nanoengineered hydrogel showed its effectiveness in bone tissue regeneration by combining osteoimmunomodulatory and antibacterial activities. The nAg/HNTs/GelMA formulation displayed fairly good cytocompatibility with human periodontal ligament stem cells (hPDLSCs) and macrophages while improving hPDLSC differentiation to osteogenic lineage in an inflammatory surrounding. Compared to HNTs/GelMA, nAg/HNTs/GelMA composition modulated the osteoimmune microenvironment in a better way and reduced gram +^ve^ and gram −^ve^ bacterial contamination both in-vitro and in-vivo rat cranial bone defects. The overall results show the effective role of nAg/HNTs/GelMA hybrid hydrogels in dealing with infected bone defects. In another study, Xin and co-workers embedded MBGN into GelMA hydrogel by an amide reaction to construct a hybrid membrane that can act as a substitute for periosteum [127]. This GelMA membrane strengthened by an inorganic constituent formed through inorganic and organic co-crosslinking resulted in a double network that showed good mechanical properties that supports blood vessels and neobone formation. The in-vivo studies also revealed its potency in hastening mature new lamellar bone formation. Another similar approach used osteoconductive GelMA hydrogel made up of bioactive hollow nanoparticles (BHPs) for bone regeneration [298]. The higher surface area of porous nanoparticles restricted the swelling characteristics of hydrogels after their incorporation. The in-vivo studies using the rat femur model showed the potential of hollow nanoparticle incorporated GelMA in recovering a large 5 mm segmental bone defect and neo tissue formation within 12 weeks.

Naturally occurring hydroxyapatite (HA) incorporated microfabricated GelMA platforms were exploited for modular tissue engineering to mimic native osteons, which are basic structural units of mature bones [126]. These biofabricated scaffolds consist of a double-ring structure with human umbilical vascular endothelial cells (HUVEC) encapsulated inner ring and human osteoblast-like cells (MG63s) encapsulated outer ring represents blood vessel tubule and bone respectively. Studies specify that the addition of HA lowered the swelling properties of nanofunctionalized hydrogel while enhanced the mechanical properties as compared to GelMA. Even though the mechanical properties of these systems do not satisfy the requirements for their clinical application, still they exhibited better cell growth and higher expression of osteogenesis and angiogenesis-related genes after differentiation marking their future application as tunable constructs for regenerating tissues.

**Fig. 12:**
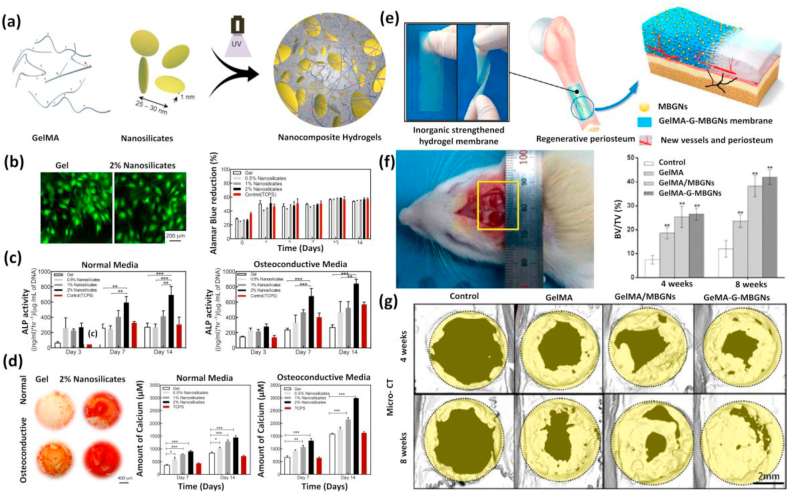
**(a)** Schematic showing the formation of nSi/GelMA hydrogel platform. **(b)** Osteogenic differentiation of MC3T3 osteoblastic cells on GelMA and nSi/GelMA hydrogel surfaces. Cell attachment and proliferation were determined and quantified on day 3 by live/dead imaging and Alamar blue assay. **(c)** Quantification of the ALP activity of cells and normalized based on the dsDNA amount present at different time points. **(d)** Optical images of alizarin red-stained hydrogel surfaces after 14 days of culture confirming the deposition of calcium salts. Reproduced with permission from Ref. [266] Copyright © 2015 American Chemical Society. (e) In-vivo bone regeneration using GelMA-M-BGN hydrogel. **(f)** Photo showing critical calvarial defect model in rat skull. **(g)** Micro-CT results of calvaria defect specimens showing higher new bone coverage area in GelMA/MBGNs and GelMA-G-MBGNs groups as compared to GelMA and empty groups at 4 and 8 weeks. Reproduced with permission from Ref. [127] copyright © 2017 American Chemical Society.

Previous studies thus highlight the vital role of inorganic components in regenerating bones by regulating mechanical properties and cellular differentiation. Except for inorganic components, some biodegradable organic components have also been introduced to impart specific functions into the GelMA hydrogel. For instance polylactic acid, a biodegradable polyester was introduced into 3D printed GelMA constructs consists of bioactive GNPs through filament freeform fabrication technique for regenerating diseased bones [299]. It was observed that the polylactic acid-reinforced GelMA platforms displayed a modulus even much higher than that of mandibular bone. The ADSCs encapsulated within this 3D functional construct displayed excellent viability, proliferation, and higher osteogenic gene expression on differentiation. On the other hand, a few investigators have taken the benefit of using non-cellular components such as ECM that structurally resemble natural tissues. It was reported that the addition of a natural ECM might facilitate bone regeneration [[300], [301], [302], [303], [304]]. The utilization of decellularized extracellular matrix (dECM) has been adapted due to their exceptional cell-supportive nature. Engineering bone tissues through an endochondral pathway using dECM have expanded interests over previous years. Biologically derived cartilage-derived matrix (CDM) particle incorporated GelMA hydrogel developed by Visser and co-workers is one such example [305]. The presence of CDM particles stimulates the differentiation of MSCs encapsulated inside the nanocomposites to chondrogenic lineage. This approach disclosed the promising role of biodegradable hydrogels in endochondral ossification and the engineering of customized bone grafts. Recently a novel strategy for bone regeneration was adopted by integrating black phosphorus nanosheets (BPN) into GelMA hydrogels [306]. The nanomaterials entrapped inside a photopolymerizable hydrogel and highly positively charged biodegradable arginine-based unsaturated poly(ester amide) show sustained release of inorganic phosphorus ions which hastens the biomineralization process by competent calcium ion absorption. The nano-engineered hydrogel adopts BMP and runt-related transcription factor (RUNX2) signaling pathway regulated by calcium ions which promoted the osteogenic potential of human dental pulp stem cells (hDPSCs). BPN hydrogels also improved the mechanical properties of the resulting hydrogel and thereby facilitating the optimum substrate properties for effective bone regeneration. Although nano-biomaterial-oriented bone tissue engineering has advanced a lot, modulating 3D stem cell differentiation deprived of exogenous components needs to be encountered. Engineering bioactive hybrid hydrogels with nSi were one such novel attempt that showed its ability to modulate stem cell differentiation in a 3D microenvironment even in the absence of additional supplements [266] (Fig. 12). After nSi incorporation, the mechanical properties of hydrogels increased four-fold as compared to the pure hydrogels possibly due to the strong electrostatic interactions which enhance several physicochemical and biological properties. Studies also revealed that the addition of 2D-nSi to ADSCs and hMSCs could bring osteogenic differentiation without using any osteoinductive factors such as BMP or DEX. Bioactive nSi composites thus show strong promise for use in a range of bone regeneration applications [181,[307], [308], [309]].

Many nanocomposites were examined actively for their potential use in soft tissue engineering but these hydrogels cannot often mineralize thereby limiting their usage in engineering bone tissues [285,[310], [311], [312]]. Recent studies explored the significance of developing new hydrogels that can mineralize. Being the vital component for bone tissue engineering, the incorporation of inorganic calcium phosphates and bioglasses into the hydrogel was of higher priority. The inorganic materials thus support mineralization by acting as nucleation sites and recovers the mechanical properties of the composite material [117,181,313,314] [[315], [316], [317], [318]]. The most accepted strategy is to generate nucleation sites by physiological mineralization and surface modification for the calcification of biomaterials. In a study, Liu et al. have reported tri-layered scaffolds fabricated using GelMA and nHA by extrusion bioprinting [185]. The physicochemical and biological properties of these scaffolds were found suitable for restoring the defects of diseased cartilaginous and subchondral bone tissues. The in-vivo studies on rabbit osteochondral defects using the fabricated constructs resulted in the regeneration of new tissues in the defect site with a smooth surface, good integrity with surrounding tissue, highly deposited cartilage-specific ECM with an abundant presence of collagen-II. This study demonstrates the favorable role of nHA/GelMA multilayer constructs in engineering osteochondral defects for rendering patient-specific 3D biomaterials [[319], [320], [321]]. Inspite of the availability of various implantable biomaterials and constructs for bone tissue engineering, the tunable mechanical properties and injectable nature of GelMA hydrogels mark their implications in treating irregular bone tissue defects.

### 4.2 Advanced platforms for skin tissue

The hydrogel wound dressing is considered as best wound treatment technique because of its excellent biocompatibility, moisture resistance, and ability to activate immune cells to promote wound healing [[322], [323], [324], [325], [326], [327]]. Polymeric biomaterials that exhibit tissue adhesiveness have emerged as a smart substitute for conventional sutures to facilitate wound closure. Out of the several natural tissue adhesives developed so far, gelatin-based adhesives have plenty of advantages because of their cytocompatibility, biodegradability, and less antigenicity [[328], [329], [330], [331], [332]]. Previous studies on GelMA gave more attention to the enhancement of mechanical properties, but the adhesive properties of hydrogels intended for engineering soft tissues were not well considered. The adhesiveness of soft hydrogels is a crucial factor that determines the efficacy of wound healing as it permits the migration of cells [[333], [334], [335], [336]].

Many GelMA-based materials were reported to show tissue adhesiveness. However, due to the poor mechanical properties of GelMA, various functional nanomaterials were mainly added to improve the mechanical properties. Assmann and colleagues developed a highly elastic adhesive hydrogel that could act as tissue sealants [332]. The developed sealant possessed biomechanical properties similar to native lung tissues and was applied for the closure of critical lung leakages. Similarly, Sani et al. reported a visible light cross-linkable adhesive GelMA hydrogel (GelCORE) for corneal repair [337]. The biophysical properties of this GelCORE hydrogel were controlled by adjusting the GelMA prepolymer concentration and crosslinking duration alone. Preclinical studies conducted on rabbit corneal defects showed superior re-epithelialization of cornea stroma by GelCORE. The effective and fast recovery of stromal defects was promising in clinical situations and has many advantages as it is a cell free approach. Some researchers also utilized interface-interaction brought healing and adhesiveness of GelMA for sutureless defect repair. A hydrogen bond-driven GelMA-based double network platform was thus developed by treating GelMA with a polyphenolic compound that acts as a hydrogen bond provider [338]. Apart from increasing the mechanical properties the hybrid hydrogels also possessed excellent tissue adhesiveness. This self-healing and superelastic hydrogel showed effectiveness in sutureless skin repair. Together with the self-healing and adhesive property of GelMA itself, the nanomaterials added enhances mechanical properties and bioactivity, and also enable the structure and chemistry more similar to the native matrix, ultimately helping the tissue regeneration.

**Fig. 13:**
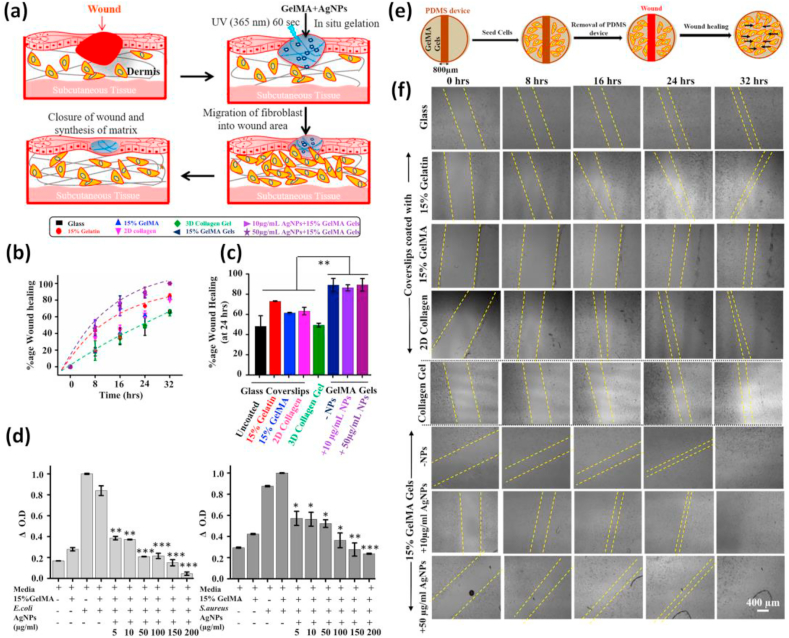
**(a)** Graphical representation showing the mode of action of AgNP/GelMA hybrid hydrogels. **(b)** Percentage of wound closure quantified as a function of time **(c)** Percentage of wound healing quantified after 24 h **(d)** Antibacterial action of 15% AgNP/GelMA formulation against *E. coli* and *S. aureus* characterized by quantifying the change in absorbance. **(e)** Schematic model of in-vitro wound healing scratch assay performed on GelMA using a PDMS device. **(f)** Shows time-dependent dynamics of wound healing experiments performed on glass, collagen, GelMA with varying AgNP content. Reproduced with permission from Ref. [217] copyright © 2019 American Chemical Society.

Studies using AgNP entrapped soft GelMA hydrogels illustrate the suitability of the hydrogel platform in motivating the healing of wounds by improving compatibility and supporting cell viability and thus allowing fast migration of fibroblasts [217]. The Ag^+^ released from the hydrogel also concurrently exhibits antibacterial activity. It was shown that the NIH 3T3 fibroblasts cultured on these substrates grow and multiply extensively on soft 15% GelMA hydrogels compared to other sets. AgNPs incorporated within the GelMA matrix even at a higher concentration of 150 μg/ml showed no significant toxicity, which agrees to the optimum Ag^+^ ion concentration required to destroy both gram +^ve^ and gram -^ve^ bacterial strains (Fig. 13). The sustained release of Ag^+^ ions thus enables tuning of AgNP concentration without considerably affecting the cell viability at different time points. Presently, the need for natural polymer-based hemostatic systems is on arise due to the drawbacks of conventional clinical surgical sealants which often lack the expected efficiency [[339], [340], [341], [342], [343]]. Compared to the synthetic surgical sealants where biodegradation is of main concern the remarkable properties of natural ones could be retained or tuned easily. Certain nanoengineered GelMA hydrogels were also known to exhibit the ability for managing bleeding and initiate blood clotting. The tissue adhesiveness and injectability of GelMA were almost similar or better than that of synthetic ones. A visible light crosslinked hybrid hydrogel developed by Rajabi et al. is one such example [344]. This novel hemostat comprised of thiol functionalized gelatin (Gel-SH), GelMA as the main polymeric components, and polydopamine coated laponite (PD-LAP) as the functional nanomaterial. The GelMA/Gel-SH/PD-LAP hybrid hemostat results through the Michael reaction between Gel-SH and GelMA, as well as the covalent interactions, arise from PD-LAP. The tissue adhesiveness, swelling behavior, dynamic properties, and potential for preventing bleeding improved significantly after the addition of PD-LAP. Moreover, it also improved the structural integrity of nano-biomaterial. The tough bio-adhesive hydrogel supported attachment and spreading of L929 fibroblasts which form a structural network by synthesizing ECM and collagen protein required for healing. Studies also confirm that GelMA/Gel-SH/PD-LAP significantly decreased the blood clotting time as compared to synthetic adhesives. In another study, Rehman et al. reported a GelMA composite hydrogel based on rGO with pro-angiogenic effects for chronic skin wound repair [345]. This porous biomaterial showed excellent wound contraction during in-vitro studies. The cell proliferation and migration promoted by rGO in GelMA/rGO composites increased the blood vessel formation in the chicken embryo model as compared to GelMA alone, signifying this carbon-based hybrid platform could be used for angiogenic therapeutics.

Liposome/GelMA nano-formulation recently synthesized by Yu and co-workers was another futuristic approach towards skin tissue engineering [346]. This system acted as a carrier hydrogel for releasing pro-healing chemokine stromal cell-derived factor (SDF-1α) in a controlled manner to induce the migration of MSCs which further exposes the role of MSCs towards wound healing and escorts the improvement of immunomodulatory and therapeutic delivery approaches for future clinical wound healing applications. Recently there is an increased interest in developing various bio-ink formulations by incorporating functional nanomaterials into GelMA for fostering skin regeneration. A low-concentration bio-ink developed by incorporating cellulose nanofibrils (CNF) within GelMA also extends its potential application towards dermal tissue engineering [268]. Even at lower concentrations, these hydrogels supported the proliferation of 3T3 fibroblasts as compared to the plain CNF hydrogel. The major properties required for GelMA-based advanced skin dressings are considered to be biodegradation, antibacterial activity, hemostasis, and adhesiveness.

### 4.3 Advanced platforms for cardiac tissue

**Fig. 14:**
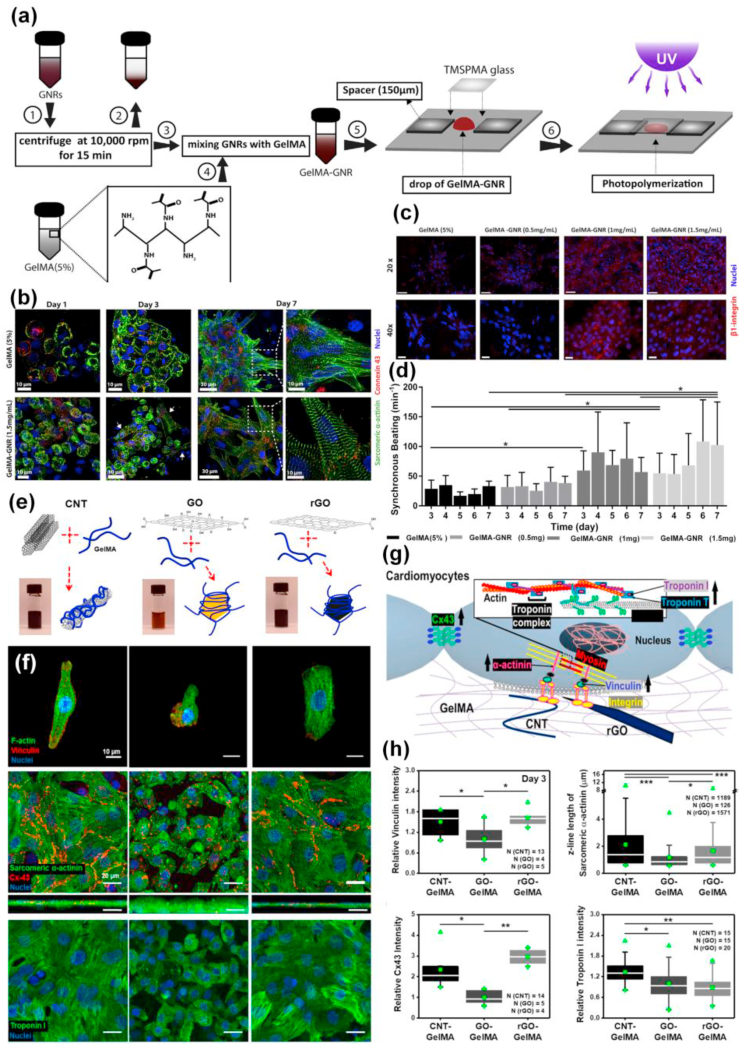
**(a)** Illustration of various steps involved in the fabrication of photo-crosslinked GNR/GelMA hybrid hydrogel **(b)** Representative confocal images showing the expression of cardiac-specific markers sarcomeric- α -actinin (green) and Cx43 (red) as analyzed by immune cytochemistry of cells cultured on 1, 3 and 7 days **(c)** Immunostaining images of cell surface receptor integrin- β1 for both pure GelMA and GNR/GelMA hybrid hydrogels at different concentrations on day 7, signifying the presence and distribution of cell adhesion units. **(d)** Stable beating behavior of cardiomyocytes on GNR/GelMA hydrogels as evaluated by synchronous beating frequency from day 3 to day 7 of growth. Reproduced with permission from Ref. [122] copyright © 2016 Elsevier. **(e)** Representation showing hybrid hydrogels resulted from CNT, GO, and rGO with GelMA **(f)** The immunofluorescent images of cardiomyocytes cultured on CNT, GO, and rGO/GelMA showing expressions for f-actin/sarcomeric α-actinin/troponin (green), vinculin/Cx-43 (red), and nuclei (blue) for 5 days of culture. **(g)** Schematic diagram depicting the role of important proteins required for maturation of cardiomyocytes. **(h)** Indicates relative intensity of vinculin, Cx-43, troponin-I, and z-line length of sarcomeric α-actinin for cardiomyocytes cultured on CNT, GO, and rGO/GelMA for 5 days. Reproduced with permission from Ref. [361] copyright © 2019 American Chemical Society.

Hydrogels with unique mechanical properties support cardiac cells mechanically to deposit ECM and form new tissues. Cellular functions and viability can be enhanced by tuning the physicochemical properties of hydrogels suitable for the regeneration of the damaged heart tissues. Rapidly gelating nanocomposite hydrogels and implantable adhesive hydrogels have thus been widely used in cardiovascular clinical therapy [347,348]. Hybrid hydrogels derived from GelMA are widely used for engineering cardiovascular tissues owing to their ability to support cell attachment to the surface and its growth and spreading [[349], [350], [351], [352]]. The majority of the nano-biomaterials currently used for rejuvenating cardiac tissues possess shortcomings such as lack of proper electrical conductivity and suitable mechanical properties, the two crucial factors playing a major role in regulating cardiac cell behavior [[353], [354], [355]]. GNRs were effectively used for myocardial regeneration due to their unique features such as biocompatibility, inertness to cells, and presence of localized surface plasmon resonance (LSPR), an optical phenomenon that plays a vital role in the conductivity of gold-based nanomaterials [[356], [357], [358], [359]]. This thus improves the electrical communication between neighboring cells and further improves cell adhesion and proliferation. Navaei et al. fabricated an advanced electroactive hydrogel by incorporating GNR into GelMA to develop functional cardiac tissue constructs for engineering cardiac tissues [122]. The GNR/GelMA hybrid gel unveiled superior electrical and mechanical characteristics as compared to pure GelMA. Chiefly, the GNR/GelMA hybrid hydrogels retained a higher number of cardiomyocytes with a better cytoskeletal organization. Studies confirmed that apart from matrix stiffness, the presence of GNR is the key factor behind this improved cell attachment and retention on the hydrogel matrix. Well-organized cardiac tissue layers formed by the integrin-mediated interaction of hydrogels and cells were also confirmed by higher expression of specific cardiac markers such as sarcomeric-α-actinin and connexin 43 (Fig. 14a–d). Being electroconductive GNR/GelMA hybrid platforms also enabled cell-to-cell signaling and propagation of electrical signals as indicated by expressions of Cx43 gap junctions and in time calcium signaling between cardiac cells exposed to GNRs of higher concentration. These improvements resulted in highly functional heart tissue constructs which precisely improved contraction of cardiac tissues with lower excitation threshold.

Nanoengineered hydrogels made by incorporating rGO into the GelMA matrix (rGO/GelMA) also possess enhanced electrical conductivity and superior mechanical properties [141,264,360]. The rGO addition resulted in the formation of extra pores which benefits the effective exchange of nutrients and waste products throughout the hydrogel utilizing diffusion. Further studies also revealed that rGO/GelMA composites could act as a supporting matrix for reconstructing damaged cardiac cells since it exhibits better biological activities and promotes maturation of 3T3 cells and primary cardiomyocytes compared to pure GelMA [361] (Fig. 14e–h). Cardiomyocytes cultured on rGO/GelMA hydrogel sheets displayed resilient contractility and rapid impulsive beating rate compared to those cells cultured on pristine GelMA and GO incorporated hydrogel sheets (GO/GelMA) of comparable concentration and mechanical stiffness. Thus the durable contractile activity in rGO/GelMA bio-constructs also offers the possibility of utilizing these systems for applications such as in-vitro drug analysis. Besides its use as an injectable formulation, nano-engineered GelMA could be patterned using several microfabrication techniques to prompt the vascularization of engineered cardiac tissue constructs or to direct the alignment of cardiac cells for the functions. Such miniaturized patient-specific constructs called “heart on a chip” are favorable platforms for future clinical trials.

### 4.4 Advanced platforms for neural tissue

**Fig. 15:**
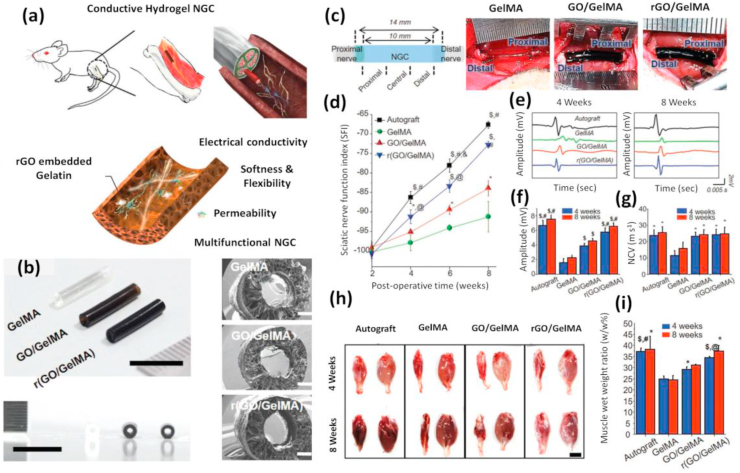
**(a)** Schematic drawing of a multifunctional conductive rGO/GelMA engineered hydrogel-based NGC possessing tunable mechanical properties, conductivity, and permeability intended for peripheral nerve regeneration. **(b)** Photos showing a side view and cross-section of individual nerve conduits with their respective scanning electron micrographs showing inner morphology. **(c)** In vivo grafting of NGCs into a sciatic nerve defect gap. **(d)** SFI for various sets at preset time points. **(e)** Evaluation of electroactivity measurements of compound motor action potential (CMAPs) for different fixed autografts and NGCs. **(f)** Onset-to-peak amplitude and **(g)** NCV for each group. **(h)** Pictures showing muscles and **(i)** Wet weight ratio of muscles for each group. Reproduced with permission from Ref. [265] copyright © 2020 John Wiley and Sons.

Nerve tissue regeneration is an advanced and fast-developing area that assures replacement or repairment of injured, diseased nerve tissues or treating major neurological disorders that are difficult to rectify employing a normal clinical approach [[362], [363], [364]]. Among the various biomaterials developed and used so far, injectable hydrogels and 3D bioprinted tubular nerve guidance conduits (NGCs) are in the frontier and are proved to be excellent candidates for promoting regeneration of injured nerve fibers across damaged peripheral nerves. NGCs are actively used for culturing and differentiating neural stem cells for central (CNS) and peripheral nervous system (PNS) regeneration [[365], [366], [367], [368]]. Considering the typical drawbacks of biomaterials used for reconstructing nerves such as biocompatibility, biodegradability, biomechanical properties, permeability, and surface properties, utilizing polymeric NGCs supports the growth and proliferation of neurons [[369], [370], [371]]. Hybrid GelMA-nanomaterial platforms were widely researched for their performance as NGCs. Recent studies conducted by Park et al. focused on the expansion of multifunctional NGCs based on rGO and GelMA for repairing peripheral nerve injury [265] (Fig. 15). The rGO/GelMA hydrogel is fabricated by chemically crosslinking GelMA and GO followed by chemical reduction. The so formed rGO/GelMA hydrogels exhibited better mechanical properties, electrical conductivity, flexibility as well as pervious nature. The in-vitro studies showed that rGO/GelMA composites promoted neuritogenesis of PC12 neuronal cells compared with GelMA and non-reduced GO/GelMA hydrogels. The in-vivo studies using a 10 mm peripheral defect-sized sciatic nerve injury model indicate that rGO/GelMA NGCs greatly improved nerve regeneration, specified by measuring the increase in muscle weight and evaluating sciatic nerve function (SFI) or nerve conduction velocities (NCV). Reports also say that rGO/GelMA platforms have extended applications in the regeneration of other electroactive tissues such as muscles or cardiac cells [154,360,361,372].

Different approaches to fabricate NGCs have been introduced in recent years to overcome the typical limitations related to nerve grafting [[373], [374], [375], [376]]. Different natural and synthetic prototypes were also introduced based on techniques such as bioprinting [377,378], electrospinning [379,380], etc which closely mimics ECM topography. In one study a customized drug embedded nerve conduits were fabricated using GelMA functionalized with poly(ethylene glycol)- poly(ε-caprolactone) or (MPEG-PCL), a copolymer with excellent biocompatibility which forms a nano-formulation [381]. The overall fabrication was assisted using digital light processed 3D bioprinting technology [[382], [383], [384]]. The tailored hydrogel conduit offers an appropriate physical microenvironment for nerve fiber elongation and the continual release of an entrapped drug, XMU-MP-1 which causes the nuclear localization of yes-associated-protein (YAP). This protein is known for its downstream effects on the Hippo pathway which upregulates YAP target genes such as GTGF and CYR61 which results in the enhanced growth and migration of Schwann cells which controls myelin elongation and thus it promotes the regeneration of peripheral nerve defects. The in-vivo electrical activity measurements and histological studies based on the sciatic nerve defect model showed increased peripheral myelination and functional recovery of nerves after the incorporation of XMU-MP-1 in hybrid NGCs. In short, this study was indeed a new approach in the regeneration of peripheral nerve defects by utilizing a biochemical signaling pathway. Fang et al. engineered a polyester-based NGC by using electrospun rGO, GelMA, and PCL nanofibers [264]. The entrapped rGO improved the electrical properties and biocompatibility of biomaterial after introducing it to GelMA/PCL matrix. It was observed that the properties of these nanofibers could be altered by varying rGO concentrations. NGCs considerably improved the spreading of Schwann cells and programmed epithelial-mesenchymal transition (EMT) related gene expression even at lower concentrations of rGO (0.25 and 0.5 wt%). The in-vivo studies disclosed functional recovery of rats after implanting NGCs without any electrical stimulation which indorsed nerve regeneration and thus safeguards the promising role of rGO/GelMA/PCL hybrid nanofibers as conductive substrates for peripheral nerve tissue engineering. The nerve grafts based on GelMA have recently been replaced by the 3D printed nerve conduits where resorbable and degradable GelMA based bio-ink is the key technology.

## 5 Nano-inspired GelMA bio-ink for miniaturized 3D tissue constructs

The 3D bioprinting arose as an innovative approach that utilizes a computer-aided process to fabricate cell-laden constructs of defined geometries that support soft or hard tissue regeneration or alternate for organ replacements [[385], [386], [387], [388], [389], [390], [391]]. Self-healable photoelastomeric bio-inks were found to be promising printable formulations for clinical studies [392]. Tunable molecular design strategies have facilitated optimal material properties like self-healing extending the application to bioactuators and hydrogel-based prosthetic devices. Owing to its excellent biological performance, photocrosslinkable GelMA and its derivatives are also reported as smart bio-inks for 3D bioprinting. (Table 2) summarizes various nano-engineered GelMA bio-inks developed so far for fabricating miniaturized tissue constructs.

**Table 2:** Summary of various nano-engineered GelMA bio-inks used for fabricating bioprinted tissue constructs.

Bio-ink	Properties of constructs	Cell type	Applications	Reference
*GNR/GelMA*	⁃ Improved electrical conduction between cardiac cells and endorsed their functional recovery ⁃ Low viscosity helps high-density cell integration. ⁃ Improved cell-cell communication, promotes harmonized contraction of constructs	CM/CF	3D functional cardiac tissue constructs	[418]
*LPN/GelMA*	⁃ Enhanced shape fidelity retention and interconnected microarchitecture within the bio-printed fibers ⁃ Controllable viscoelastic properties and the ability to spatially control bioactive molecule release ⁃ Supported cell maturation, retention of growth factor, and it's delivery	HBMSC	Functional cell-instructive nanocomposite bio-ink for 3D bone tissue regeneration.	[399]
*Sr/GelMA*	⁃ Enhanced viscosity, excellent shape fidelity, scaffold shape retention, ⁃ Improved osteogenic differentiation	hMSC	Extrusion-based 3D bioprinting of permissive cell-laden scaffolds	[405]
*κCA/nsi/GelMA (NICE bio-ink)*	⁃ Excellent printability for complex (taller and higher aspect ratio structures), extensive cell-laden constructs for tissue engineering. ⁃ Custom bioprinted scaffolds and tissue-engineered implants of high structural fidelity and mechanical stiffness.	hMSC	Nanoengineered bio-ink for the fabrication of mechanically rigid and elastomeric 3D constructs.	[190]
*MSN/BMP-4/GelMA*	⁃ Satisfactory printability, mechanical stability, and biocompatibility. ⁃ Promoted osteogenic differentiation and accelerated bone repair in diabetic bone defects.	BMSC and RAW 264.7	Extrusion-based 3D bioprinted scaffolds for diabetic bone repair.	[411]
*HAMA/CNC/GelMA*	⁃ Reinforced bio-ink with excellent printability ⁃ Defines shape and provides satisfactory structural support to the 3D printed constructs.	ATDC5 cells	Cellulose nanocrystal reinforced bio-ink for improved structural Integration	[430]
*PLGA NF/GelMA*	⁃ Bio-ink with improved viscosity ⁃ Supports fibroblast proliferation	NIH3T3 cells	Nanofiber fragment reinforced bio-ink for 3D bioprinting.	[437]
*DECM/nanoclay* */GelMA*	⁃ 3D scaffolds with better printability and biocompatibility	Primary hepatocytes	Better organ-specific biomaterial ink for primary hepatocytes cultivation	[426]

**Fig. 16:**
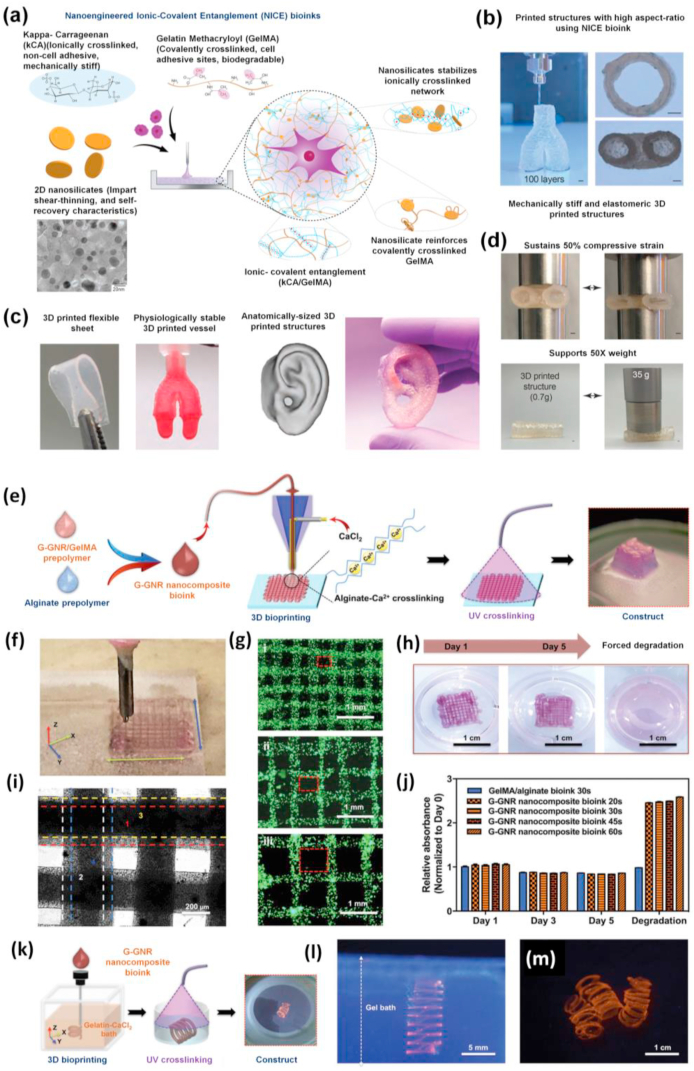
Tough, elastic, and highly printable NICE bio-ink formed by bond reinforcement mechanisms **(a)** Dual reinforced polymeric network formed from GelMA and kCA through ionic–covalent entanglement via nSi. **(b)** Self-supported hydrogel constructs printed using NICE bio-ink exhibit a high aspect ratio and print fidelity. **(c)** Various bioprinted constructs resulted from NICE bio-ink which closely resembles anatomical structures **(d)** The bioprinted structures exhibit superior rigidity and elastic properties and could support more than 50-times of their actual weight. Reproduced with permission from Ref. [190] copyright © 2018 American Chemical Society. **(e)** Illustration showing the bioprinting process using GNR/GelMA bio-ink. **(f)** Printing is illustrated on a cartesian co-ordinate system and its view through a microscope showing a stacked arrangement of different layers. **(g)** 3D printing of constructs with diverse internal grids. GNR/GelMA contained green beads displays printed fibers. **(h)** Bioprinted constructs show stability in culture medium up to 5 days and also show its forced degradation in an enzymatic environment **(i)** The absorbance studies of cell culture medium revealed the GNR release upon degradation **(j)** Illustrating bioprinting using the 3D embedded nanocomposite bio-ink. **(k)** Shows the spiral construct in the support bath composed of gelatin and CaCl_2_ after printing. **(l)** Bioprinted constructs were collected from the supporting bath without damaging it and incubated in the culture medium. Reproduced with permission from Ref. [418] copyright © 2017 John Wiley and Sons.

Nanoengineered ionic covalent entanglement bio-ink (NICE), a novel smart bio-ink developed by Chimene and colleagues for 3D bone bioprinting is one of a kind [190]. This bio-ink formulation comprised of GelMA, kappa-carrageenan (kCA), a linear hydrocolloid polysaccharide, and 2D-nSi (Fig. 16a–d)**.** The presence of two independent entwined polymer chains in this bio-ink conjugated themselves via a distinct cross-linking mechanism allows precise control over its characteristics. The presence of nSi enhances the mechanical stiffness and tissue adhesiveness by exerting reversible electrostatic interactions within the hydrogel chains. Being a printable hydrogel formulation this could be utilized for the fabrication of various mechanically robust cellularized structures [190]. Further, it has added advantages such as low cost and enzyme degradability. The occurrence of improved mechanical properties of resultant constructs is ascribed to the stress distribution and energy dissipation achieved through the disruption of an ionic network while the flexibility of the chemically cross-linked network remains the same [[393], [394], [395]]. These ionic and covalent interactions in NICE bio-ink are considered to be the reason for their elastomeric characteristics under mechanical loading without compromising mechanical features. Cell-induced remodeling over 60 days was well demonstrated using hMSC encapsulated within the constructs to induce endochondral differentiation even in the lack of osteoinductive agents. The studies also elucidated the bioactive role of nSi, a key component of NICE bio-ink for stimulating the endochondral differentiation [176,[396], [397], [398]]. The osteoinductive bio-ink can induce differentiation to mineralize the ECM in a growth factor-free environment. This approach represents a significant advance in hybrid bio-ink technology and will be demonstrated to be an expedient tool for the bioprinting of large, complex tissue structures by conjoining synergistic interactions between nanomaterials and hydrogels. Another inorganic nanomaterial-based functional cell-instructive bio-ink was made using laponite (LPN) and GelMA [399]. This system displayed superior properties such as retention of shape fidelity and meshed pore morphology within the bioprinted fibers. Human bone marrow stromal cells (HBMSCs) encapsulated using the bio-ink supports osteogenesis in absence of osteoinductive components such as DEX due to the favorable microenvironment for differentiation. Studies showed that HBMSCs encapsulated in LPN/GelMA proliferated over 21 days of culture compared to pure GelMA. Ex-vivo studies using bioprinted constructs by chick extraembryonic membrane model confirmed exceptional integration of the material in the vascular chick embryo after growing it for 7 days. LPN/GelMA composites loaded with angiogenic growth factor, VEGF also showed considerably higher permeation of blood vessels than the control groups. The cell supportive features of this bio-ink composition such as cell proliferation, retention of growth factor, etc. are thus beneficial for the regeneration of hard and soft tissues.

Improved cytocompatibility and bioactivity are the most essential requirements in nanomaterials intended to be used in developing new bio-ink. Many inorganic ions are capable of inducing cellular differentiation. So there exists massive promise for these ions for effective use in bone regeneration. The role of strontium (Sr) in the remodeling and engineering of bone tissues has been reported in recent years [[400], [401], [402], [403], [404]]. A low concentration bio-ink made up of GelMA and strontium-carbonate nanoparticles, (Sr/GelMA) was used for extrusion-based bioprinting of shape fidile scaffolds [405]. Sr/GelMA bio-ink retained their mechanical and physical properties. Rheology analysis of samples showed a significant variation in GelMA viscosity after the addition of Sr nanoparticles. The bioprinted scaffolds displayed excellent shape dependability as evinced by a well-defined interconnected pore structure which supports cell growth for the long term. Apart from imparting desired mechanical properties, the addition of Sr also resulted in enhanced osteogenic differentiation of hMSCs as confirmed by higher expressions of osteogenic markers such as osteocalcin (OCN), collagen type-I (Col I), and higher levels of ALP. Previous studies also report the role of Sr ions in stimulating the activity of osteoblasts while inhibiting the osteoclasts thereby highlighting the potential of Sr/GelMA bio-ink to enhance both the physical and biological performance of printed constructs towards their clinical application in regenerating bone tissues. It is known that bioprinted constructs loaded with cells and osteoinductive factors could improve cellular activities even in an inflammatory microenvironment and further promotes bone regeneration [304,[406], [407], [408]]. It is also known that the sustained release of bioactive factors can trigger the differentiation of cells [409,410]. Recently Sun et al. fabricated MSN-based scaffolds for regenerating bones in diabetic conditions [411]. The porous scaffolds containing BMSCs, RAW264.7 macrophages, and BMP were developed based on a modified bio-ink composed of GelMA, gelatin, 4-arm poly (ethylene glycol) acrylate, a biocompatible polymer, and MSN (GelMA/Gelatin/PEG/MSN). The addition of MSNs meritoriously enhanced the physicochemical properties of scaffolds. Besides this BMP-4 loaded onto MSNs showed a sustainable release for enduring efficacy. In presence of BMP, the RAW264.7 cells undergo polarization and result in M2 macrophages. The anti-inflammatory factors released by these macrophages could decrease inflammation in the microenvironment. The osteoinductive factors released from the MSN and M2 macrophages simultaneously excite the differentiation of BMSCs to osteogenic lineage and further accelerated diabetic bone repair.

The regeneration of cardiac tissues using nanoengineered hydrogels also profusely progressed due to the new insights of converging microscale technologies with stem cell biology. Even though a lot of noteworthy studies have been done focused on developing bio-engineered cardiac constructs or patches for functional tissue replacement, a clinically applicable engineered cardiac structure representing the morphological, physiological, and functional properties of natural myocardium remains the challenge [22,[412], [413], [414], [415], [416], [417]]. Surfactant modified GNR (C-GNR) coated with GelMA (G-GNR) incorporated into GelMA matrix resulted in a nanocomposite bio-ink formulation used for the 3D bioprinting of functional cardiac constructs [418]. (Fig. 16e-m) The presence of G-GNRs in G-GNR/GelMA bio-ink doesn't influence the cytocompatibility of constructs. Moreover, it improved the electrical transmission between cardiac cells and supported their functional development in the 3D-printed cardiac constructs. The comparative studies conducted using bio-constructs made from G-GNR/GelMA and GelMA/Alginate showed higher expression of intercellular channel proteins such as Cx-43 in G-GNR/GelMA. Besides this, the synchronized contractile behavior exhibited by G-GNR/GelMA was much higher than that in constructs made of GelMA/Alginate. The role of G-GNR in controlling the proliferation of cardiac fibroblasts (CFs) by varying the mRNA expression of proteins involved in contractile action which maintains the cardiomyocyte to cardiac fibroblast ratio (CM: CF ratio) was also supposed to be the main reason behind the improved contractile performance. It is envisioned that this nanocomposite bio-ink is applicable in creating a more customized and biomimetic 3D printed cardiac construct with contractile properties for drug testing, at the same time having the potential for implantation in an affected area in the heart following acute myocardial infarction. This practice could also contribute to rejuvenating other electrogenic tissues, such as the spinal cord, brain tissue, and skeletal muscle [419,420]. Recently tissue-specific ECM, made using the decellularization process has been proposed in several approaches for tissue and organ reconstruction [[421], [422], [423], [424], [425]]. Compared to many existing organ-specific decellularized extracellular matrix (DECM) bio-ink formulations developed so-far, recently developed GelMA, DECM, and nanoclay based bio-ink contained the highest proportion of DECM (around 75%) which improved its biomechanical and cell supportive properties [426]. This nanocomposite system was used for primary hepatocyte cultivation due to its unique printability.

3D bioprinting of soft tissue constructs was always a challenge for biomedical researchers since it lacks the desired mechanical properties [[427], [428], [429]]. Advanced printing technologies provided options for solving the problems by utilizing polymeric structural supports. Hybrid printing technology characterized by simple fabrication and integrated structural features exploits the association of diverse bio-inks having the same crosslinking mechanism. Mechanically reinforced cell-laden nanocomposite bio-ink developed by Fan et al. is an example [430]. The formulation consists of GelMA, HAMA, and cellulose nanocrystals (CNC). The incorporated CNCs reinforce the bio-ink by exerting electrostatic and dipole-dipole attraction between the hydrogel composition. This bio-ink composition offered more flexibility towards the construction of large cell-laden assemblies with better structural integrity. Recent approaches also attempt to reinforce bio-inks for improving the biomechanical properties and dimensional stability [352,[431], [432], [433], [434], [435], [436]]. GelMA modified with poly(lactic-co-glycolic acid) nanofiber fragments (PLGA-NF), a copolymer macromolecule is an example [437]. Studies show that fibroblasts encapsulated within PLGA-NF/GelMA constructs showed enhanced cellular activities as compared to the control groups. Customized patient-specific soft tissues fabricated via bioprinting using programmed modeling and optimum parameters make use of these nanofiber fragments as an artificial ECM marking its importance for future tissue engineering applications. Briefly, the merits of GelMA hydrogel in bioprinting are remarkable in terms of tunable material properties within synthetic methods offering a 3D platform for a wide spectrum of tissues. On the other hand, developing bioactive inks with optimal cellular behaviors, exceptional printability, and shape fidelity is still a major challenge to find clinical availability.

## 6 Conclusion and future perspectives

The nano-structured hydrogels of succeeding generations have got potential to transform or replace the existing systems for various tissue engineering applications. The superior characteristics owned by multi-functional hybrid systems, which lack in their hydrogel counterparts enable them for various applications. Being a dominant hydrogel formulation of recent times, the future direction on GelMA-nanomaterial platforms certainly includes balanced optimization in all aspects says physical, chemical as well as biological. Next-generation nano-engineered GelMA is being progressively evaluated for its various clinical applications. Even though the non-functionalized nanomaterial and GelMA interactions could result in useful material characteristics, the resultant hybrid composites lack control over many key properties including stimuli responsiveness and physiological degradation. To overcome these drawbacks different strategies have been introduced in which modifying GelMA hydrogel network by incorporating multifaceted nanomaterials of various types is the most common approach. Our research group also recently focused on developing similar systems by functionalizing antioxidant nanomaterials such as cerium oxide (CeO2) using macromolecules such as dendrimers which would interact with the hydrogel physically or chemically and resulted in multi-responsive scaffolds of better stiffness and microarchitecture. Nowadays more efforts are also made in designing hydrogels with long-term biocompatibility that will aid desired biological behavior. The reviewed literature proposes that the development of novel synthetic routes, functionalizing strategies, and fabrication technologies for GelMA-nanomaterial hybrid systems will continue in the coming years and more priority will be given to research for its personalized applications. Microfabrication approaches for knowing the cell-nanophase communication is one such attempt. Microscale technologies are emerging as a prevailing technology to solve the many existing challenges in tissue engineering. Studies conducted using CNT reinforced hybrid microgels and hyperbranched polyesters have shown the effective utilization of microscale technologies for cell entrapping on pre-defined patterns resulting in different functional tissues of interest. Engineered GelMA hydrogel holds a groundbreaking role in hastening the progress in clinical therapeutics by converging biological and bio-fabrication approaches. Apart from the advances, there are many challenges linked to the clinical translation of photocrosslinkable hydrogels like GelMA. The most important factor is the conversion of various synthetic strategies towards successful regeneration methods and procedures by accepting good manufacturing practices (GMP) while conserving the envisioned capacity for tissue regeneration. Furthermore, evaluations on nano-functionalized constructs are mandatory before the clinical applications to limit the concerns related to biosafety. Certain nanocomposite hydrogels could be applicable only in culturing specific cell types (eg- cardiac cells, nerve cells, etc.) compared to non-functionalized GelMA hydrogel platforms, due to the intrinsic material characteristics. Consequently integrating universal functionalities is also important to attain tissue engineering applications in a broader range. GelMA-based hydrogel constructs have not been completely applied for clinical applications due to limitations concerning their biosafety associated with using UV radiations used for crosslinking which can damage cellular DNA by oxidation and also could result in sudden inheritable changes. Assessing the biosafety for both cell-free and cell encapsulated GelMA-nanomaterial platforms is thus of utmost importance to ensure safe and effective usage for its future biomedical applications.

## Declaration of competing interest

The authors declare no competing financial interest.
